# Targeting β-catenin signaling for therapeutic intervention in *MEN1*-deficient pancreatic neuroendocrine tumours

**DOI:** 10.1038/ncomms6809

**Published:** 2014-12-17

**Authors:** Xiuli Jiang, Yanan Cao, Feng Li, Yutong Su, Yanli Li, Ying Peng, Yulong Cheng, Changxian Zhang, Weiqing Wang, Guang Ning

**Affiliations:** 1 Shanghai Clinical Center for Endocrine and Metabolic Diseases, Shanghai Key Laboratory for Endocrine Tumors, Rui-Jin Hospital, Shanghai Jiao-Tong University School of Medicine, Shanghai 200025, China; 2 Laboratoire Génétique Moléculaire, Signalisation et Cancer, CNRS, UMR5201, Faculté de Médecine, Université Claude Bernard Lyon1, Centre LEON-BERARD, 69373 Lyon, France; 3 Laboratory of Endocrinology and Metabolism, Institute of Health Sciences, Shanghai Institutes for Biological Sciences (SIBS), Chinese Academy of Sciences (CAS) & Shanghai Jiao-Tong University School of Medicine (SJTUSM), Shanghai 200025, China

## Abstract

Inactivating *MEN1* mutations are the most common genetic defects present in sporadic and inherited pancreatic neuroendocrine tumours (PNETs). The lack of interventional therapies prompts us to explore the therapeutic approach of targeting β-catenin signalling in *MEN1*-mutant PNETs. Here we show the *MEN1-*encoded scaffold protein menin regulates phosphorylation of β-catenin. β-catenin signalling is activated in *MEN1*-mutant human and mouse PNETs. Conditional knockout of β-catenin suppresses the tumorigenesis and growth of *Men1*-deficient PNETs, and significantly prolongs the survival time in mice. Suppression of β-catenin signalling by genetic ablation or a molecular antagonist inhibits the expression of proproliferative genes in menin-null PNETs and potently improves hyperinsulinemia and hypoglycemia in mice. Blockade of β-catenin has no adverse effect on physiological function of pancreatic β-cells. Our data demonstrate that β-catenin signalling is an effective therapeutic target for *MEN1-*mutant PNETs. Our findings may contribute to individualized and combined medication treatment for PNETs.

## Abstract (web summary)

*MEN1* gene encodes menin, a nuclear scaffold protein that regulates transcription and is often inactivated in pancreatic neuroendocrine tumours (PNETs). Here Jiang *et al.* show that *MEN1*-driven PNET development involves activation of β-catenin, and that β-catenin deletion ameliorates the disease.

## Graphical abstract

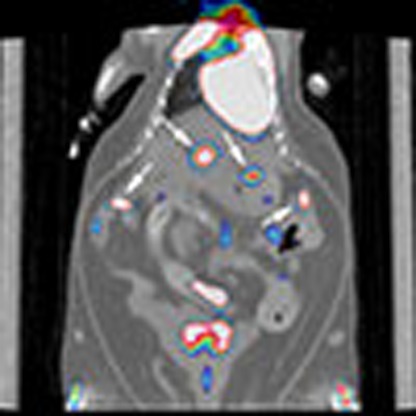

Pancreatic neuroendocrine tumours (PNETs) arise from the pancreatic endocrine cells and are broadly classified into functional (hormone-producing) and nonfunctional tumours. The majority of PNETs are sporadic. Some PNETs occur in familial syndromes, such as multiple endocrine neoplasia type 1 (MEN1), von Hippel–Lindau syndrome and tuberous sclerosis. The prevalence of PNETs has substantially increased in recent decades, making PNETs the second most common malignancy of the pancreas. The prognosis of patients with PNETs that have metastasized is poor, with a survival of only 1–3 years^1^,^2^,^3^,^4^.

Inactivating *MEN1* mutations are the dominant genetic defects present in benign and malignant, sporadic and inherited PNETs. Germline mutations in *MEN1* are the main cause of familial PNETs. A loss of heterozygosity, which was observed in PNETs with inactivating mutations in *MEN1*, results in the deficiency of the encoded protein menin in tumours^5^,^6^. A recent genetic study demonstrated that 44.1% of sporadic PNETs harbor inactivating somatic mutations in the *MEN1* gene^7^. The therapeutic targets and important mechanisms for *MEN1*-deficient PNETs are still largely unknown. The development of potent therapeutic approaches for *MEN1*-deficient tumours is the greatest challenge in reducing the morbidity and mortality of patients with PNETs.

Menin, a highly conserved and ubiquitously distributed scaffold protein, plays functional roles in multiple physiological and pathological processes. Menin can interact with a number of transcription factors and recruit chromatin-modifying proteins, including MLL, HDACs and Sirt1, to regulate the expression of tissue-specific target genes^8^,^9^,^10^,^11^,^12^. In mouse pancreatic β-cells, the deletion of *Men1* accelerates cell proliferation and leads to the formation of functional PNETs (insulinomas)^13^. Acute *Men1* deletion improved the preexisting hyperglycaemia in Streptozotocin (STZ)-induced diabetic mice^14^. In previous studies, we described the nuclear accumulation of β-catenin in *Men1*-deficient PNETs. Menin interacts with β-catenin and transports it out of the nucleus via nuclear–cytoplasmic shuttling, which controls the activity of Wnt/β-catenin signalling^15^.

The activation of the Wnt/β-catenin signalling pathway is frequently observed in several cancers, including colorectal cancer and hepatocellular carcinoma^16^. Canonical Wnt signalling induces the nuclear accumulation of β-catenin in a complex with LEF/TCF transcription factors, which regulates gene transcription^17^. Cytosolic β-catenin can be phosphorylated and targeted for proteolysis by a complex of proteins. CK1 phosphorylates β-catenin at Ser45, which primes β-catenin for subsequent phosphorylation by GSK-3β at Ser33, Ser37 and Thr41 refs 18, 19. Activating mutations at the phosphorylation sites result in the stabilization of β-catenin and tumorigenesis. Various Wnt/β-catenin signalling pathway members have been detected at different stages in the mouse and human pancreas^20^. Previous studies reported the functions of Wnt/β-catenin signalling in the development and replication of pancreatic cells, but the correlation between β-catenin and the physiological functions of pancreatic β-cells remains controversial^21^,^22^,^23^,^24^,^25^. Determination of the roles of β-catenin in tumorigenesis of PNETs needs to be resolved. The systematic *in vivo* evaluation of targeting Wnt/β-catenin signalling in *Men1*-deficient PNETs might contribute to the development of novel therapeutics.

Here we investigate the effects of blocking β-catenin signalling in PNETs of β-cell-specific *Men1* knockout mice. The conditional knockout of β-catenin in *Men1*-deficient PNETs suppresses tumorigenesis and significantly improves hypoglycemia and the survival rate in mice. Antagonizing β-catenin signalling by the small molecule inhibitor PKF115-584 in *Men1*-deficient PNETs suppresses tumour cell proliferation *in vitro* and *in vivo*. The deletion of β-catenin is tolerated for normal β-cell functions. These findings may provide novel therapeutic approaches for *Men1*-deficient PNETs.

## Results

### Menin promotes the phosphorylation of β-catenin

**Figure 1:**
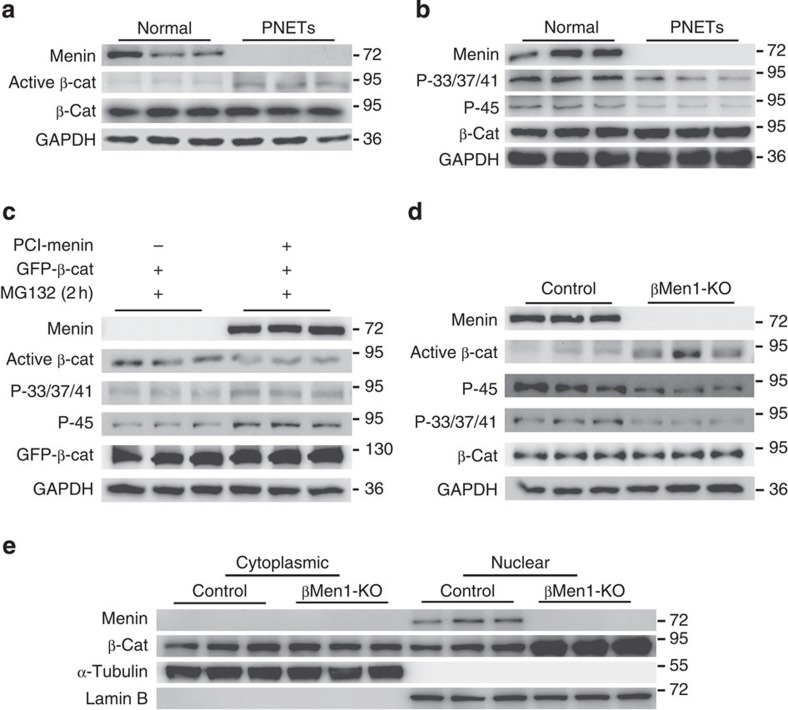
Activation of β-catenin signalling in *MEN1*-mutant human and mouse PNETs. (**a**) Western blot analyses of menin, β-catenin and active β-catenin in human *MEN1*-mutant PNETs and normal islets (*n*=3). (**b**) Western blot analyses of phospho-33/37/41-β-catenin (P-33/37/41) and phospho-45-β-catenin (P-45) in human *MEN1*-mutant PNETs and normal islets (*n*=3). (**c**) Western blot analyses of active β-catenin, phospho-33/37/41-β-catenin (P-33/37/41) and phospho-45-β-catenin (P-45) in 293T cells transfected with green fluorescent protein–β-catenin (GFP–β-catenin) and menin or vector control. The transfected cells were treated with the proteasome inhibitor MG132 (25 μM) for 2 h before the cells were harvested. The data shown represent three independent experiments. (**d**) Western blot analyses of active β-catenin and phosphorylated β-catenin in the menin-null islets from 12-month-old βMen1-KO mice and normal islets from control mice (*n*=3 for each group). (**e**) Western blot analyses of cytoplasmic and nuclear β-catenin in the pancreatic islets isolated from 12-month-old βMen1-KO and control mice (*n*=3 for each group).

Alterations in β-catenin phosphorylation affect the activity of β-catenin signalling. Disturbance of the phosphorylation process results in the tumorigenesis of many tissues^16^. To evaluate the effect of menin on β-catenin phosphorylation, we examined the phospho-β-catenin levels in *MEN1*-mutant PNETs. The significant increasing of active β-catenin and decreasing of phospho-β-catenin on residues Ser33, Ser47, Thr41 and Ser45 were observed in menin-null tumours compared with normal human islets (Fig. 1a,b). The active β-catenin is detected by the antibody specific for the dephosphorylated β-catenin on Ser37 or Thr41. The *in vitro* experiments showed that the phospho-β-catenin on residues Ser33, Ser47, Thr41 and Ser45 were markedly elevated upon menin overexpression (Fig. 1c). Conditional *Men1* knockout led to a significant reduction of phospho-β-catenin and an increase in the amount of active β-catenin in mouse PNETs (Fig. 1d). Moreover, we examined the subcellular distribution of β-catenin. The data showed elevated nuclear accumulation of β-catenin in *Men1*-deficient PNETs (Fig. 1e). Taken together, these results indicate that the loss of menin facilitated the stabilization and activation of β-catenin in PNETs.

### Ablation of β-catenin inhibits menin-null tumour growth

**Figure 2:**
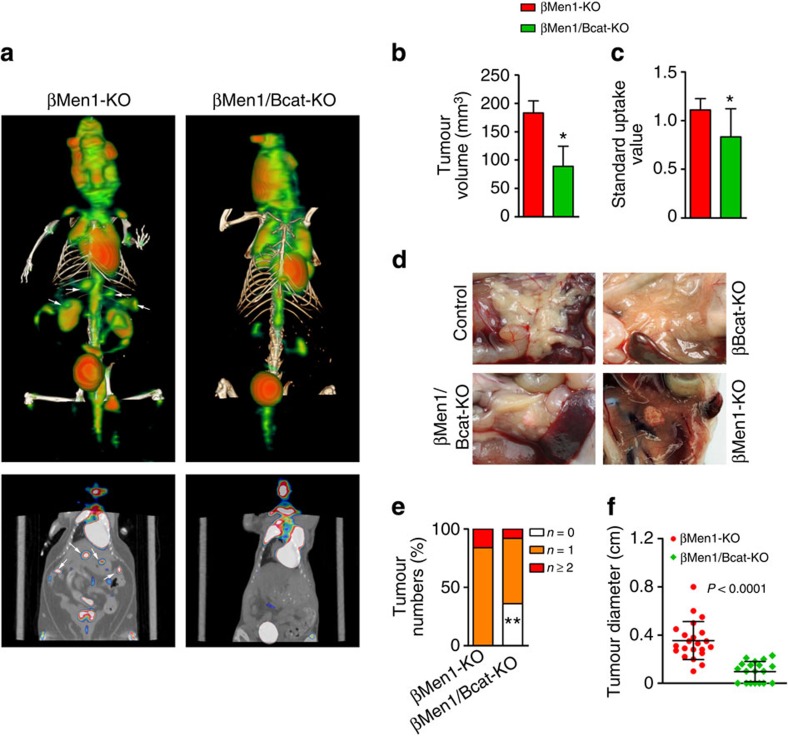
β-catenin ablation inhibits tumorigenesis of menin-null PNETs in mice. (**a**) Representative PET/CT fused images of PNETs in the βMen1-KO and βMen1/Bcat-KO mice. The arrowheads point to the tumour lesions that have high uptake of FDG. (**b**) Analysis of the average tumour volume in the βMen1-KO and βMen1/Bcat-KO mice by PET/CT (*n*=3 for each group). (**c**) Analysis of the standard uptake value of 18F-FDG in mouse PNETs by PET/CT (*n*=3 for each group). The data represent the mean±s.d., *P<0.05, Student’s *t*-test. (**d**) Gross anatomy of the pancreas and tumour burden in mouse models at the age of 15 months. (**e**) Analysis of the tumour numbers in the 15-month-old βMen1-KO and βMen1/Bcat-KO mice (*n*=25 for each group). The percentages of mice with no tumour, one or more than one tumour were analyzed. **P<0.01, Fisher’s exact test. (**f**) Analysis of the tumour diameter in the 15-month-old βMen1-KO (*n*=22) and βMen1/Bcat-KO mice (*n*=19). The average tumour diameter of the mice with multiple PNETs was plotted. For the mice that have no PNET, The tumour diameter was plotted as zero.

To investigate the functional roles of β-catenin signalling in *Men1*-deficient PNETs *in vivo*, we generated β-cell-specific menin and β-catenin double knockout mice (βMen1/Bcat-KO) and β-catenin knockout mice (βBcat-KO). These models were constructed by crossing *Men1*^*flox/flox*^, *Ctnnb1*^*flox/flox*^ mice and *Rip-Cre*^*Herr*^ mice^26^. The β-cell-specific knockout of *Men1* (βMen1-KO) leads to the development of benign insulinomas in mice^13^. The tumour lesions in age-matched βMen1-KO and βMen1/Bcat-KO mice were assessed by micro-positron emission tomography/computed tomography (micro-PET/CT) scanning. The reconstructed three-dimensional images and PET/CT-mapping images showed that the lesion numbers and tracer uptake intensity were significantly decreased in the βMen1/Bcat-KO mice compared with the βMen1-KO mice (Fig. 2a). Quantification of these alterations ascertained that the βMen1/Bcat-KO mice had markedly decreased total lesion volume and lower fluorine-18 fluorodeoxyglucose uptake than the βMen1-KO mice (Fig. 2b,c). The gross anatomy of the mouse pancreas revealed that the βMen1/Bcat-KO mice had a smaller tumour burden compared with the βMen1-KO mice (Fig. 2d). The tumour numbers in the βMen1/Bcat-KO mice were significantly decreased. More than one-third of the βMen1/Bcat-KO mice escaped from the development of tumours (Fig. 2e). The average diameter of the insulinomas in the βMen1/Bcat-KO mice was reduced >70% compared with the βMen1-KO mice (Fig. 2f).

**Figure 3:**
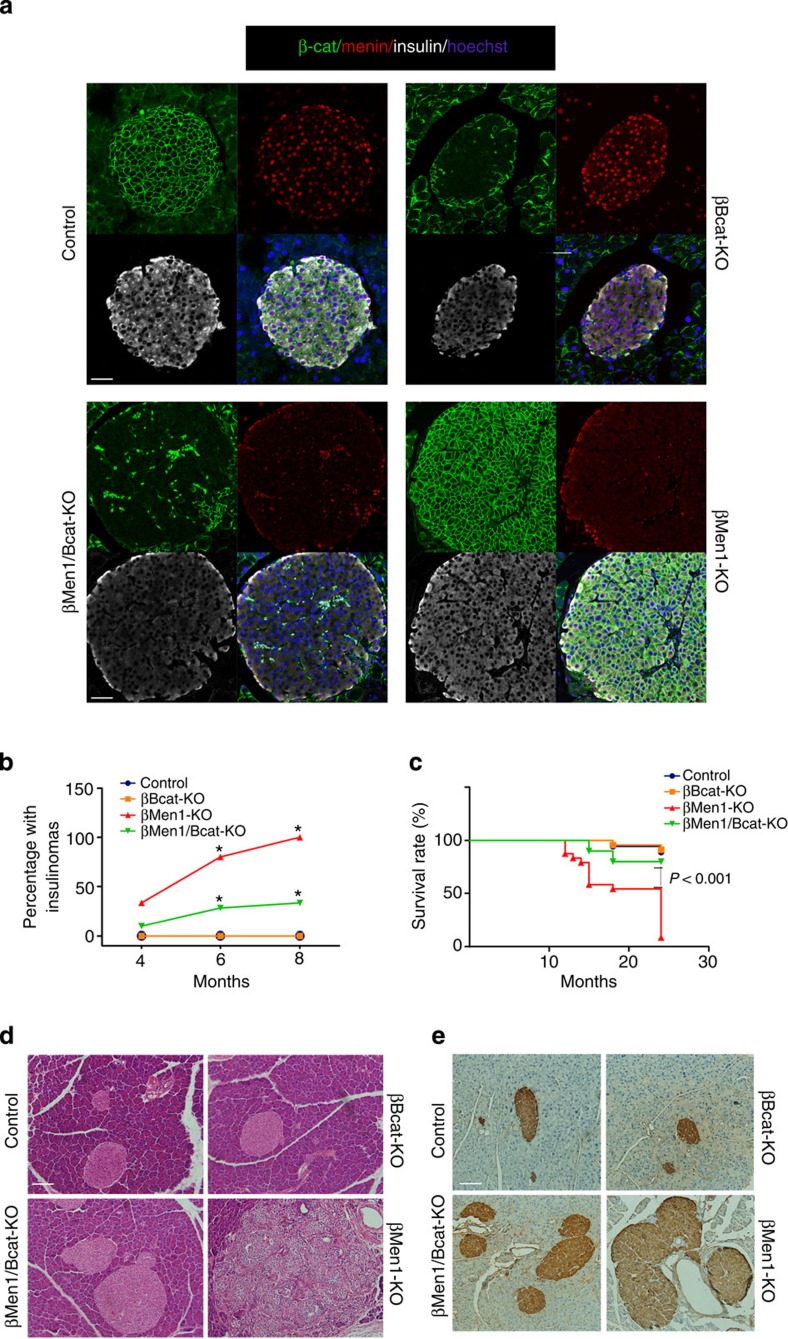
Deletion of β-catenin suppresses growth of menin-null PNETs and increases survival time. (**a**) Immunofluorescence staining of menin, β-catenin and insulin on pancreatic sections from 8-week-old mouse models. Scale bars, 50 μm. (**b**) The incidence of tumorigenesis in mouse models (*n*=18–31). *P<0.05, Fisher’s exact test. (**c**) Analysis of the survival rate of mouse models (*n*=18–24) in 2 years. *P*<0.001, Fisher’s exact test. (**d**) Hematoxylin–eosin staining on pancreatic sections from 15-month-old mouse models. Scale bars, 100 μm. (**e**) Histochemical staining of insulin on pancreatic sections from 6-month-old mouse models. Scale bars, 100 μm.

The expression of menin and β-catenin in the pancreatic islets of the mouse models were examined by immunofluorescence, Western blot and quantitative real-time reverse transcription–PCR (RT–PCR) (Fig. 3a and Supplementary Fig. 1). The histological examinations revealed that the tumour incidence of the βMen1/Bcat-KO mice was 52% lower than the βMen1-KO mice at the age of 6 months. All of the βMen1-KO mice had insulinomas at age of 8 months, while only one-third of the βMen1/Bcat-KO mice developed tumours. PNETs were absent in the control and βBcat-KO mice (Fig. 3b). The severe hypoglycemia (<1.1 mmol l^−1^) induced by insulinoma could cause death of aged βMen1-KO mice. A significant increase in survival rate was observed in the βMen1/Bcat-KO mice compared with the βMen1-KO mice. Only 8.3% of the βMen1-KO mice survived at the age of 24 months, while 80% of the βMen1/Bcat-KO mice survived. The βBcat-KO mice had comparable survival time with the control mice (Fig. 3c). Histologically, the PNETs in βMen1-KO mice exhibited a characteristic nesting and trabecular pattern, while the majority of the pancreatic islets in βMen1/Bcat-KO mice showed moderate hyperplasia (Fig. 3d,e). Malignant or metastatic PNETs were not detected by gross anatomy observation and histological tissue examination in βMen1-KO and βMen1/Bcat-KO mice.

All together, these findings suggest that the ablation of β-catenin in *Men1*-deficient PNETs inhibits tumorigenesis and tumour growth. Targeting β-catenin could significantly extend the lifespan of the *Men1*-deficient mice.

### β-catenin ablation does not affect β-cell function

To further explore whether the suppression of β-catenin impairs the normal development and function of pancreatic β-cells, we examined the islet architecture and glucose metabolism in βBcat-KO mice. Comparative histological studies showed normal development of the pancreas in the βBcat-KO mice at E18 (Supplementary Fig. 4a). The architecture of the mature pancreatic islets in the βBcat-KO mice was comparable to the control mice (Supplementary Fig. 4b). There was no significant difference in the body weight, food intake, fasting blood glucose and serum insulin levels between the βBcat-KO mice and the control mice on a normal chow diet (Supplementary Fig. 4c–f). Glucose tolerance tests revealed non-significant changes in the glucose metabolism of the βBcat-KO mice (Supplementary Fig. 4g).

We investigated the effects of β-catenin ablation on the non-neoplastic proliferation of pancreatic β-cells. The glucose metabolic changes in the βBcat-KO and control mice on a high-fat diet (HFD) for 4 months were analyzed. Chronic HFD consumption could lead to compensatory replication of the pancreatic β-cells in mice. Our results showed no difference in the gain of body weight between the βBcat-KO and control mice (Supplementary Fig. 5a). The glucose and insulin tolerance tests indicated that the glucose metabolism of the βBcat-KO mice was comparable to the control mice on long-term HFD (Supplementary Fig. 5b–d). Our data suggested that the inhibition of β-catenin signalling does not impair the physiological functions of β-cells. Targeting β-catenin in mature pancreatic β-cells would not lead to impaired glucose tolerance or diabetes.

### β-catenin KO rescues cell proliferation and hypoglycemia

**Figure 4:**
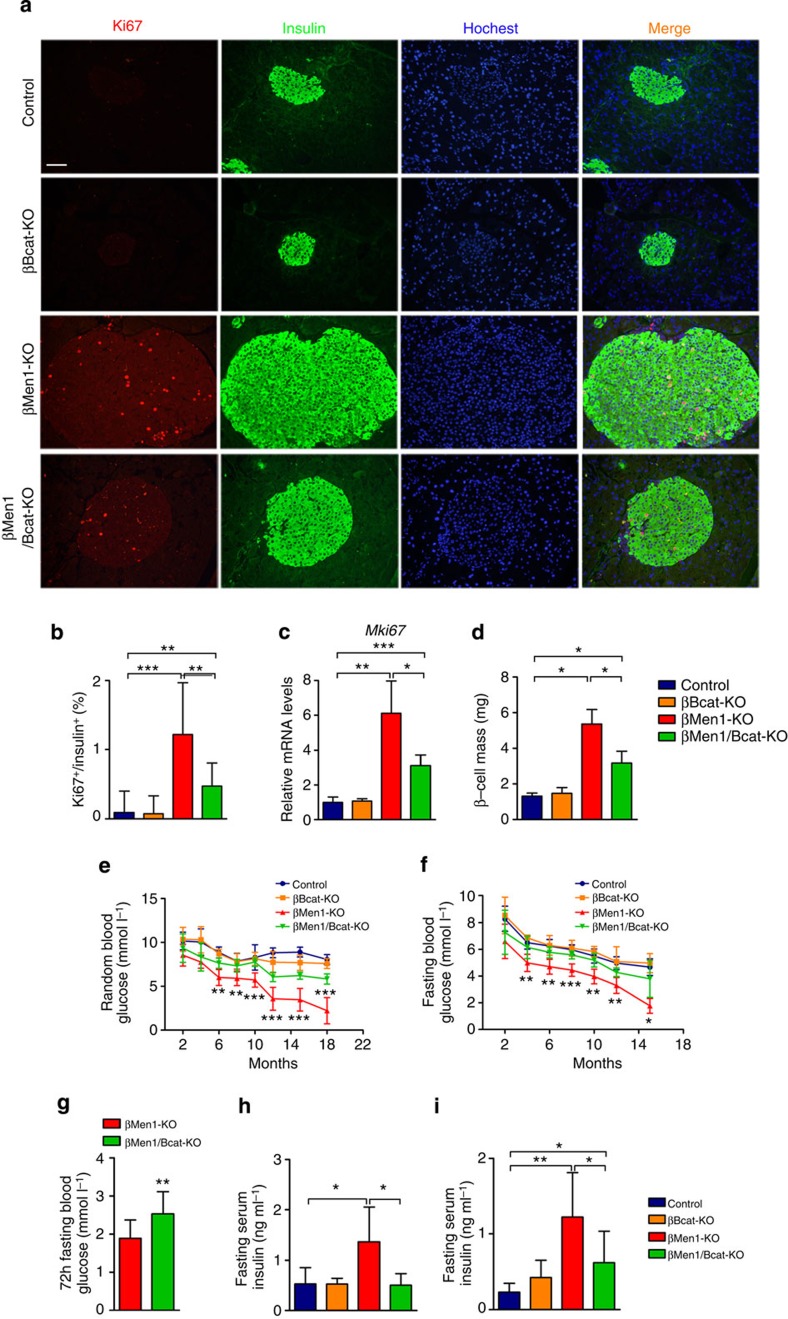
β-catenin knockout inhibits tumour cell proliferation and excessive insulin production in *Men1-*deficient mice. (**a**,**b**) Immunofluorescence staining and analysis of Ki67 on pancreatic sections from 6-month-old mouse models (*n*=3). Scale bars, 100 μm. (**c**) Quantitative PCR analysis of *Mki67* expression in the islets isolated from 6-month-old mouse models (*n*=4–6). (**d**) Quantification of pancreatic β-cell mass from 4-month-old mouse models. (**e**,**f**) Random (**d**, *n*=8) and fasting (**e**, *n*=6–8) blood glucose levels in mouse models. The mice were fasted for 16 h. (**g**) Seventy-two hours fasting blood glucose levels in the 12-month-old βMen1-KO and βMen1/Bcat-KO mice (*n*=13 for each group). (**h**,**i**) Fasting serum insulin levels in mouse models at the ages of 10 months (**h**, *n*=6) and 15 months (**i**, *n*=6–8). The mice were fasted for 16 h. The data represent the mean±s.d., *P<0.05, **P<0.01, ***P<0.001, Student’s *t*-test.

To evaluate the pancreatic cellular proliferation in the mouse models, Ki67 staining analysis was performed on the pancreatic sections to visualize cell division. We detected a 12-fold increase in the cell proliferation rate in the insulinomas of βMen1-KO mice. β-catenin knockout led to a twofold reduction of Ki67-positive menin-null β-cells. The βBcat-KO mice had an indistinguishable proliferation rate of β-cells compared with the controls (Fig. 4a,b). The messenger RNA (mRNA) levels of *Mki67* were significantly downregulated by β-catenin deletion in the *Men1*-deficient islets (Fig. 4c). The β-cell mass had a fourfold increase in βMen1-KO mice compared with control mice, whereas β-catenin ablation markedly decreased the β-cell mass in the βMen1/Bcat-KO mice (Fig. 4d). These data suggest that β-catenin blockade could partially rescue the expansion of *Men1*-deficient β-cells.

The increasing β-cell mass and development of insulinomas result in severe hyperinsulinemia and hypoglycemia in βMen1-KO mice. The blood glucose levels of the βMen1-KO mice continuously decreased after 2 months of age. The aging βMen1-KO mice suffered lethal hypoglycemia caused by the growth of insulinomas, whereas no significant changes of blood glucose were observed in aging βBcat-KO mice. The blockade of β-catenin markedly improved the hypoglycemia in the *Men1*-deficient mice (Fig. 4e,f). The long-term (72 h) fasting blood glucose examinations showed that the βMen1-KO mice suffered severe hypoglycemia, whereas the βMen1/Bcat-KO mice had significant improvement (Fig. 4g). The examinations of fasting serum insulin levels showed that the β-catenin deletion rescued the hyperinsulinemia in *Men1*-deficient mice (Fig. 4h,i). These data showed that the changes of insulin levels and β-cell mass were consistent in mouse models, which indicated that the β-catenin deletion mainly inhibited menin-null tumour expansion.

**Figure 5:**
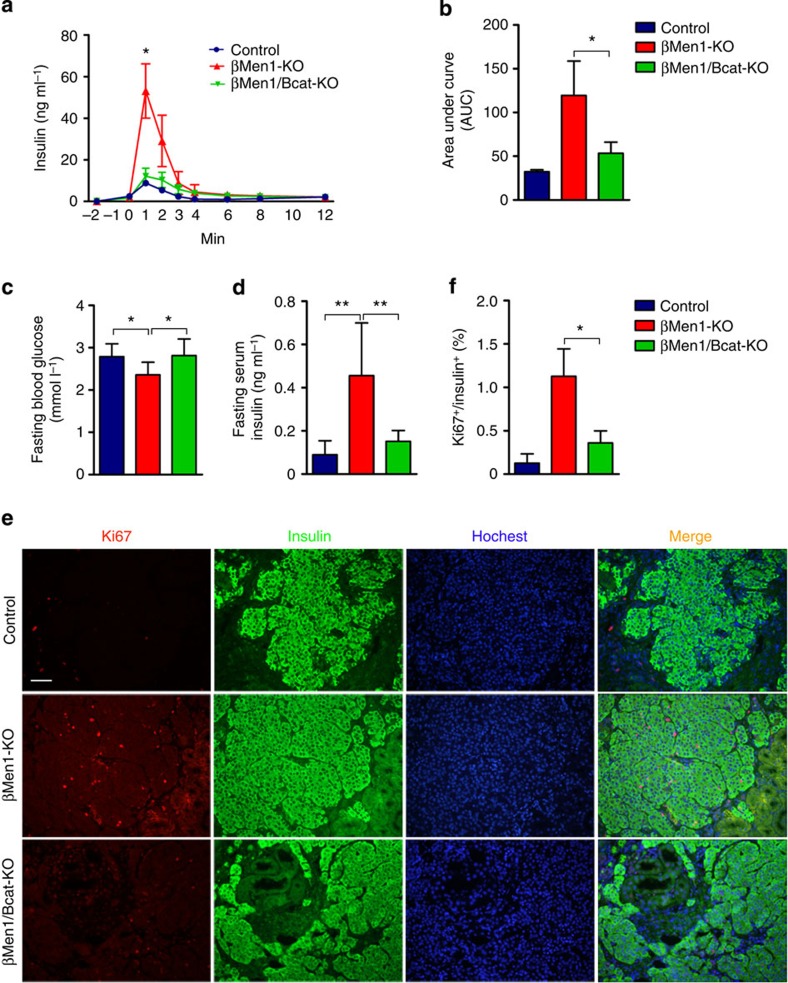
β-catenin knockout decreases excessive insulin secretion of *Men1*-deficient PNETs in mice. (**a**) Insulin levels in the pancreatic perfusion buffer in the *ex vivo* pancreatic glucose (16.7 mmol l^−1^) perfusion tests (*n*=3–7). (**b**) Analysis of the area under curve (AUC) of the insulin levels in the *ex vivo* pancreatic glucose perfusion tests (*n*=3–7). (**c**,**d**) 24-h fasting blood glucose (**c**) and serum insulin levels (**d**) in the STZ-induced diabetic mice transplanted with islets from control, βMen1-KO and βMen1/Bcat-KO mice (*n*=6–8). (**e**,**f**) Analysis of Ki67 staining on the sections of transplanted grafts from mouse models (*n*=3 for each group). The data represent the mean±s.d., *P<0.05, **P<0.01, Student’s *t*-test. Scale bars, 100 μm.

To determine whether the effects of β-catenin blockage were specifically restricted in the β-cells, we conducted *ex vivo* pancreatic perfusion and islet transplantation experiments. The pancreatic insulin release of the βMen1-KO mice was remarkably higher than the control mice. The pancreas from the βMen1/Bcat-KO mice exhibited comparable insulin release curves to the control mice (Fig. 5a,b). We transplanted islets isolated from βMen1-KO, βMen1/Bcat-KO and control mice into STZ-induced diabetic mice. The transplantation of islets from the βMen1-KO mice resulted in significantly lower blood glucose and higher serum insulin levels in the recipient mice compared with the effects of islets from the βMen1/Bcat-KO and control mice (Fig. 5c,d). The lack of β-catenin restricted the menin-null β-cell proliferation in the islet grafts (Fig. 5e,f and Supplementary Fig. 6).

Collectively, the deletion of β-catenin could effectively inhibit menin deficiency-driven β-cell proliferation and significantly improve the insulinoma-induced severe hyperinsulinemia and hypoglycemia in *Men1*-deficient mice.

### Ablation of β-catenin inhibits proliferative gene expression

**Figure 6:**
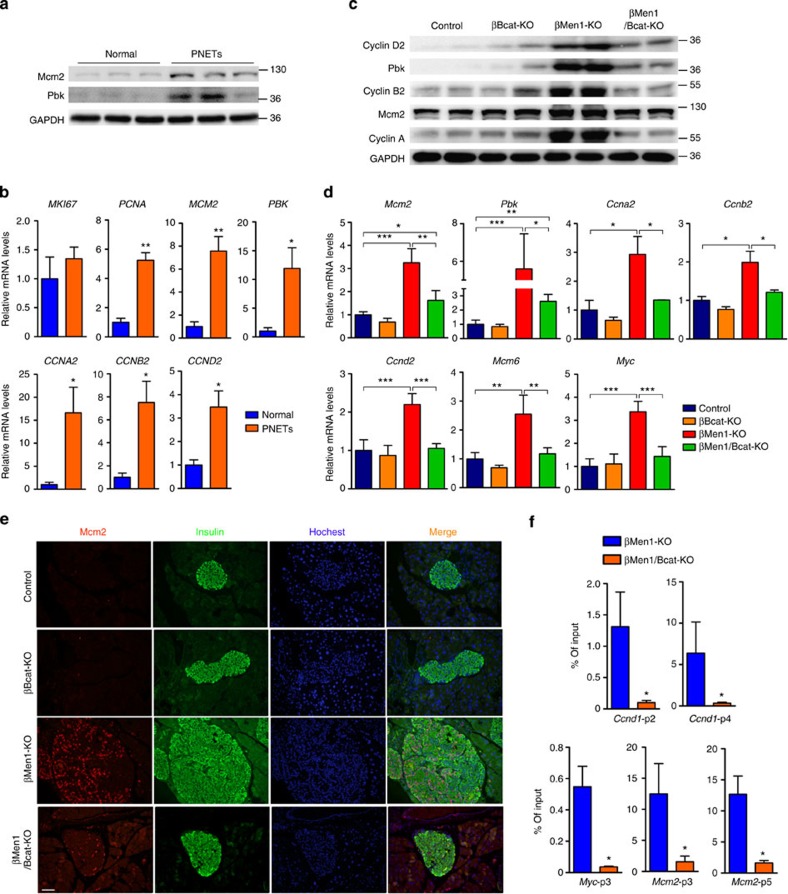
Inhibition of β-catenin suppresses proproliferative gene expression in *MEN1*-mutant PNETs. (**a**) Western blot analyses of Mcm2 and Pbk in human *MEN1*-mutant PNETs and normal islets (*n*=3). (**b**) Quantitative PCR (qPCR) analysis of *MKI67*, *PCNA*, *MCM2*, *PBK*, *CCNA2*, *CCNB2* and *CCND2* in human *MEN1*-mutant PNETs and normal islets (*n*=3). (**c**) Western blot analyses of Mcm2, Pbk, Cyclin D2, Cyclin B2 and Cyclin A in isolated islets from mouse models. (**d**) qPCR analysis of *Ccna2*, *Ccnb2*, *Ccnd2*, *Mcm2*, *Mcm6*, *Myc* and *Pbk* in isolated islets from mouse models (*n*=3–6). The data represent the mean±s.d., *P<0.05, **P<0.01, ***P<0.001, Student’s *t*-test. (**e**) Immunofluorescence staining of Mcm2 and insulin in pancreatic sections from mouse models. Scale bars, 100 μm. (**f**) ChIP assays were performed to show that β-catenin bind the promoters of *Ccnd1*, *Myc* and *Mcm2* in control and menin-null islets. The pancreatic islets were isolated from 12-month-old mouse models. Sonicated cell lysates were incubated with β-catenin antibody for protein–DNA binding detection. Triplicate qPCR reactions for each sample were performed using four primer pairs covering ~2,800 bp of the *Ccnd1* promoter, four primer pairs covering ~1,400 bp of the *Myc* promoter and six primer pairs covering ~1,500 bp of the *Mcm2* promoter. The results from 660–400 bp (p2) and 2,750–2,552 bp (p4) of upstream regions of the *Ccnd1* promoter; 1,030–885 bp (p3) of the *Myc* promoter; 712–585 bp (p3) and 1,428–1,326 bp (p5) of the *Mcm2* promoter are shown. All data are normalized against immunoglobulin-G control and expressed as percentage of input. The data represent the mean±s.d., *P<0.05, Student’s *t*-test. The data shown represent three independent experiments.

Previous studies have reported that menin deficiency results in the upregulation of multiple key genes controlling cell cycle, DNA replication and mitosis^14^,^27^,^28^. To gain insight into the underlying molecular mechanisms of the tumour suppression effect observed in βMen1/Bcat-KO mice, we screened the expression of pivotal factors, including DNA replication licensing factor minichromosome maintenance proteins (MCMs), cyclin family proteins and PBK (PDZ binding kinase), in human and mouse menin-null PNETs. The protein levels of Mcm2 and Pbk were significantly elevated in the *MEN1-*mutant PNETs compared with normal human islets (Fig. 6a). Significant upregulation of *PCNA*, *MCM2*, *PBK*, *CCNA2*, *CCNB2* and *CCND2* in *MEN1-*mutant PNETs was detected (Fig. 6b). Moreover, the protein and mRNA levels of Pbk, Mcm2, Cyclin B2, Cyclin D2 and Cyclin A were markedly increased in menin-deficient tumours and dramatically rescued to normal levels by β-catenin ablation in mice (Fig. 6c,d and Supplementary Fig. 7). The immunofluorescent staining showed that the enhanced expression of Mcm2 in the mouse *Men1*-deficient islets was significantly decreased by β-catenin ablation (Fig. 6e). Furthermore, we performed chromatin immunoprecipitation (ChIP) assay for β-catenin at the promoters of the potential target genes in the islets from mouse models. Our data show that β-catenin was recruited to the promoters of *Ccnd1*, *Myc* and *Mcm2* in control and menin-null islets. Loss of menin resulted in the enrichment of β-catenin at the promoters of *Ccnd1*, *Myc* and *Mcm2* in *Men1*-KO cells compared with the control cells (Fig. 6f). These results indicate that the activated β-catenin signalling promotes the expression of key proproliferative genes in a direct and indirect manner in *MEN1*-deficient cells. The suppression of β-catenin could repress the growth of menin-null tumours by rescuing the upregulation of proliferative genes.

### Antagonist of β-catenin inhibits tumour cell proliferation

**Figure 7:**
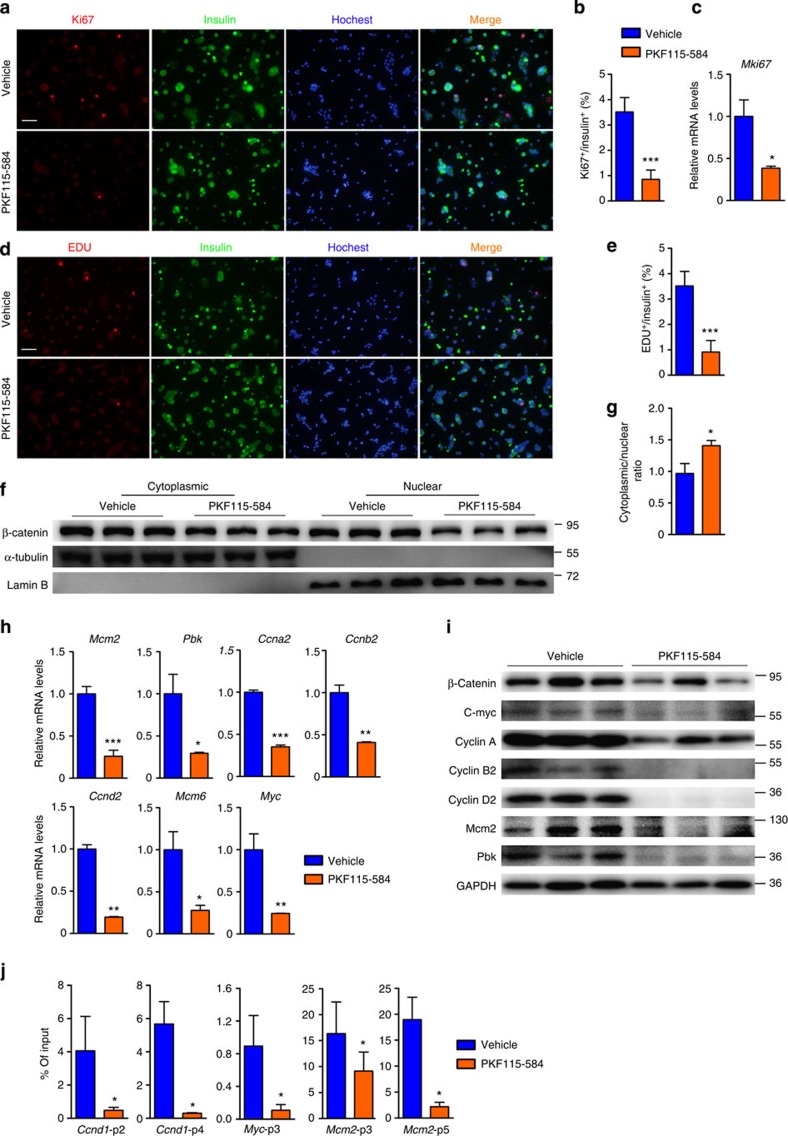
β-catenin antagonist inhibits replication of menin-null tumour cell and expression of proproliferative genes *in vitro*. (**a**,**b**) Analysis of Ki67 staining on PKF115-584 (1 mM for 16 h) or vehicle-treated dispersed tumour cells from βMen1-KO mice (*n*=3). Scale bars, 100 μm. (**c**) Quantitative PCR (qPCR) analysis of *Mki67* expression in PKF115-584- or vehicle-treated tumour cells from βMen1-KO mice (*n*=3). (**d**,**e**) Analysis of EdU staining on PKF115-584- or vehicle-treated dispersed tumour cells from βMen1-KO mice (*n*=3). Scale bars, 100 μm. (**f**,**g**) Western blot analyses of cytoplasmic and nuclear β-catenin in PKF115-584- or vehicle-treated tumour cells from βMen1-KO mice (*n*=3). (**h**,**i**) qPCR and Western blot analyses of *Mcm2*, *Pbk*, *Ccna2*, *Ccnb2*, *Ccnd2*, *Mcm6* and *Myc* expression in PKF115-584- or vehicle-treated tumour cells from βMen1-KO mice (*n*=3). (**j**) ChIP assays were performed to show the binding of β-catenin at the promoters of *Ccnd1*, *Myc* and *Mcm2* in vehicle or PKF115-584 (1 mM for 16 h) treated tumour cells from βMen1-KO mice (*n*=3). The pancreatic islets were isolated from 12-month-old βMen1-KO and βMen1/Bcat-KO mice. Sonicated cell lysates were incubated with β-catenin antibody for protein–DNA binding detection. Triplicate qPCR reactions for each sample showed the results from 660–400 bp (p2) and 2,750–2,552 bp (p4) of upstream regions of the *Ccnd1* promoter; 1,030–885 bp (p3) of the *Myc* promoter; 712–585 bp (p3) and 1,428–1,326 bp (p5) of the *Mcm2* promoter. All data are normalized against immunoglobulin-G control and expressed as percentage of input. The data represent the mean±s.d., *P<0.05, **P<0.01, ***P<0.001, Student’s *t*-test. The data shown represent three independent experiments.

To assess whether an antagonist of β-catenin signalling could suppress the expansion and excessive insulin production of *Men1-*deficient tumours, βMen1-KO mice and isolated tumour cells were treated with PKF115-584, which is a small molecule antagonist of the TCF/β-catenin complex^29^. The Ki67 staining and *Mki67* expression analysis showed that the PKF115-584 treatment inhibited the proliferation of menin-null cells (Fig. 7a–c). In addition, the 5-ethynyl-2′-deoxyuridine (EdU) proliferation assays indicated that the replication of menin-null cells was significantly suppressed by PKF115-584 (Fig. 7d,e). The treatment with PKF115-584 could decrease both cytoplasmic and nuclear β-catenin levels as reported in other systems^30^,^31^,^32^ (Fig. 7f), while the cytoplasmic/nuclear ratio of β-catenin was significantly increased (Fig. 7g). The mRNA and protein levels of the pivotal proliferative genes in *Men1*-deficient tumour cells were significantly downregulated by the β-catenin antagonist (Fig. 7h,i). Furthermore, ChIP assays for β-catenin at the promoters of *Ccnd1*, *Myc* and *Mcm2* in the PKF115-584 or vehicle-treated menin-null cells revealed that β-catenin recruitment to the promoters was attenuated by PKF115-584 treatment (Fig. 7j). These data indicate that the PKF115-584 specifically decreases β-catenin at the promoters of the target genes through β-catenin/TCF inhibition. The replication of *Men1*-deficient β-cells was significantly suppressed by PKF115-584.

**Figure 8:**
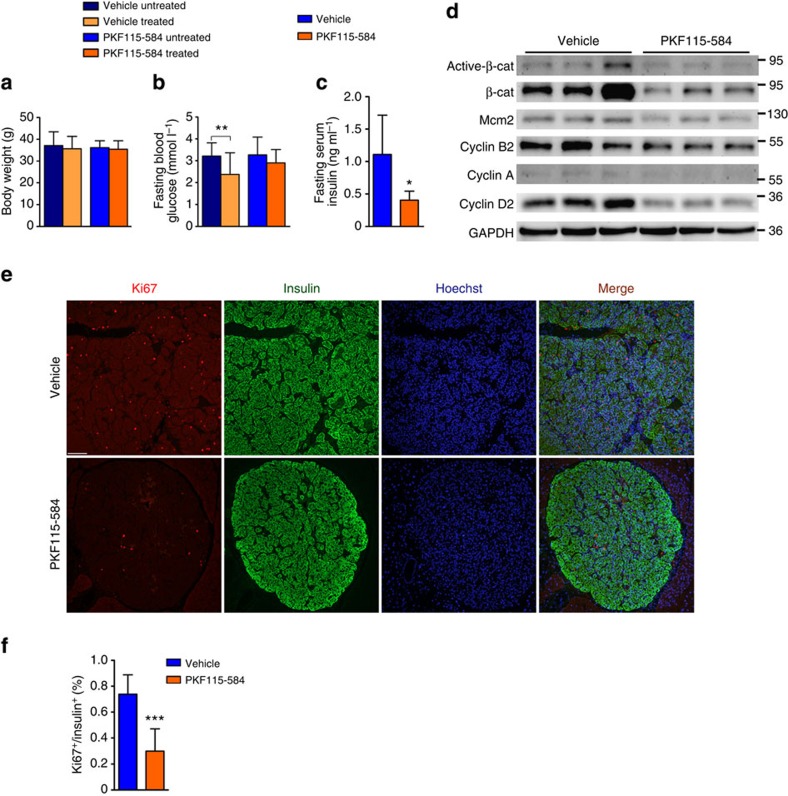
A β-catenin antagonist suppresses *Men1*-deficient tumour cell proliferation and improves hypoglycemia in mice. (**a**) Body weight of βMen1-KO mice treated with PKF115-584 (0.5 mg kg^−1^) or vehicle (*n*=7–8). The untreated and treated groups represent the beginning and end of treatment. (**b**,**c**) Fasting blood glucose and fasting serum insulin levels in βMen1-KO mice treated with PKF115-584 or vehicle (*n*=6–8). The data represent the mean±s.d., *P<0.05, **P<0.01, Student’s *t*-test. (**d**) Western blot analyses of active and total β-catenin, Mcm2 and cyclin proteins in PNETs from βMen1-KO mice treated with PKF115-584 or vehicle. The data shown represent three independent experiments. (**e**,**f**) Analysis of Ki67 staining in PNETs from βMen1-KO mice treated with PKF115-584 or vehicle (*n*=3 for each group). Scale bars, 100 μm.

Furthermore, we explored the specific effects of β-catenin antagonist on βMen1-KO mice *in vivo*. The 14-month-old βMen1-KO mice were treated with PKF115-584 (0.5 mg kg^−1^) or vehicle *in vivo* every 3 days for 8 weeks. The antagonist treatment prevented the decline in the blood glucose and reduced hyperinsulinemia compared with the vehicle group without affecting the body weight (Fig. 8a–c). The inhibition of β-catenin resulted in the downregulation of key proliferative genes in *Men1*-deficient tumours (Fig. 8d). The Ki67 staining suggested that the β-catenin antagonist suppressed cell proliferation in *Men1*-deficient PNETs (Fig. 8e,f). In addition, mice treated with PKF115-584 had a trend towards increased survival (87.5 versus 67.7%), even though the data did not reach statistical significance (*P=*0.414, Fisher’s exact test) (Supplementary Fig. 8).

Taken together, these findings indicate that the suppression of β-catenin signalling by a small molecule antagonist could effectively inhibit the *Men1*-deficient tumour proliferation and prevent hypoglycemia *in vitro* and *in vivo*.

## Discussion

The majority of patients diagnosed with PNETs present with either metastatic or regionally advanced disease. Surgery is the primary therapy for the patients with localized or metastatic PNETs. Unfortunately, the prognosis of patients with advanced and progressive PNETs is poor^1^,^2^,^3^. The limited options of effective treatment for this highly deadly disease raise the urgent need for the development of therapeutic agents and combination therapy programmes. The oral mTOR inhibitor Everolimus (RAD001) and multi-targeted tyrosine kinase inhibitor Sunitinib can significantly prolong the progression-free survival among patients with advanced PNETs^33^,^34^, proving that therapeutic interventions targeting tumour-associated signalling pathways should be the future approach for PNETs. The efficiency and efficacy of the current molecular therapy for PNETs are limited. Individualized and combined agent treatment should be further developed for patients with PNETs.

A comprehensive understanding of genetic lesions is fundamental for the development and application of molecular targeting therapies for cancer. Recently, exome sequencing of the nonfunctional PNETs revealed highly frequent *MEN1*, *DAXX*/*ATRX* mutations and alterations in the mTOR pathway genes^5^. Our group identified the hotspot *YY1* T372R mutation in 30% of insulinomas^35^. The mutations in the mTOR signalling genes, which are targets of Everolimus, only account for 15% of the nonfunctional PNETs. According to these genetic studies, the *MEN1* mutation is a proximal event that initiates PNET tumorigenesis. Genetic and pharmacologic advances have shed light on the regroup of tumours by the driver gene mutations and signalling pathways for individualized treatment. The investigations into small molecule agents against *MEN1-*deficient PNETs will contribute to efficient individualized and combined therapies.

The loss of menin in *MEN1*-mutant PNETs suggests that the downstream activating signalling in menin-deficient tumours might be a therapeutic target for PNETs. Our previous report showed that menin could interact with β-catenin and regulate the subcellular localization of β-catenin through nuclear–cytoplasmic shuttling^15^. In this study, our data indicate that menin negatively controls the phosphorylation, nuclear accumulation and activation of β-catenin via shuttling β-catenin out of the nucleus in pancreatic β-cells. Moreover, we demonstrate that the activated β-catenin signalling pathway can be targeted for therapeutic interventions of *MEN1*-mutant PNETs. Genetic ablation of β-catenin can significantly suppress the tumour formation and growth in βMen1-KO mice. The hypoglycemia and hyperinsulinemia are markedly improved in the βMen1/Bcat-KO mice and the PKF115-584-treated βMen1-KO mice. β-catenin ablation significantly prolongs the survival time of the βMen1-KO mice. Notably, majority of our data indicate that the blockade of β-catenin partially suppressed the effects resulted from *Men1* deficiency, suggesting other pathways could be involved in menin-null PNETs.

Previous studies reported that the scaffold protein menin could recruit multiple partners and regulate genes transcription in a tissue-specific manner^12^. Our results reveal that the elevated expression of several proliferative factors, including Mcms, cyclin proteins and Pbk, can be rescued by β-catenin signalling suppression in *MEN1*-deficient tumours. Activated β-catenin is recruited to the promoters and stimulates expression of *Mcm2*, *Ccnd1* and *Myc*, which is inhibited by the β-catenin small molecule inhibitor PKF115-584. These findings suggest that antagonizing β-catenin signalling would be an effective treatment for *MEN1*-mutant PNETs.

Our data demonstrate that the suppression of β-catenin had no obvious effects on development, physiological functions or the compensatory proliferation of pancreatic β-cells. The inhibition of β-catenin signalling activity is crucial for *MEN1-*mutant β-cell tumours without affecting the physiological function of β-cells. Previous studies have shown that mTOR inhibitor treatment could lead to the impairment of β-cell function and mass^36^. Our results indicate that treatment of PNETs with Wnt/β-catenin inhibitors might not result in the risk of hyperglycaemia and diabetes. However, the high doses and long-term PKF115-584 treatment *in vivo* is toxic for mice^37^,^38^. In our study, the specific effects of β-catenin antagonist would be more significant if it was suitable for prolonged treatment *in vivo*. The effects of PKF115-584 on tumor number and diameter, which should be evaluated before and after the treatment by self-control analysis in live mice, have not been assessed *in vivo*.

Previous studies have demonstrated that β-catenin signalling is an important potential target for many cancer types. Inactivating mutations in the tumour suppressor genes and activating mutations in β-catenin can lead to the abnormal activation of Wnt/β-catenin signalling and tumorigenesis^16^. Somatic mutations in *FAT1* result in Wnt activation in multiple types of cancer, including glioblastoma and colorectal cancer^39^. In endocrine-related cancers, activating mutations in *CTNNB1* are common in adrenal tumours^40^. The development of small molecules targeting β-catenin signalling for *in vivo* use is a major research interest in cancer drug discovery. PKF115-584, which can inhibit the growth of multiple myeloma cells and adrenocortical carcinoma cells, is a molecule targeting the TCF and β-catenin complex^29^,^33^,^41^. Recently, a selective porcupine inhibitor, LGK974, was examined in a phase 1 clinical trial. LGK974 can potently inhibit Wnt signalling *in vitro* and *in vivo* and suppress cell proliferation in Wnt-driven cancers^42^,^43^. Novel Wnt/β-catenin inhibitors could be used for the clinical treatment of *MEN1*-mutant PNETs in the future. The combination treatment of a β-catenin inhibitor and an mTOR inhibitor might be beneficial for PNETs harbouring *MEN1* mutations and mTOR signalling gene mutations.

Collectively, our data show that menin deficiency promotes the dephosphorylation and activation of β-catenin in *MEN1*-mutant PNETs. Targeting β-catenin signalling by a molecular antagonist or a genetic approach significantly inhibits the proliferation of *MEN1*-deficient tumours. The hypoglycemia and mortality in *Men1* knockout mice with PNETs could be markedly ameliorated by the blockade of β-catenin signalling. Our findings might provide a potent therapeutic approach for treatment of *MEN1*-mutant tumours and highlight the importance of β-catenin inhibitors in future individualized treatment strategies for PNETs.

## Methods

### Clinical samples

Frozen human PNET samples were obtained from the endocrine-related tumour bank of the Shanghai Key Laboratory for Endocrine Tumours. Normal human pancreatic islets were isolated from the donor pancreas for transplantation. The normal human islets of Langerhans used in this study were isolated according to the Edmonton protocol. Informed consent was obtained from all of the study participants. All of the protocols were approved by the Rui-jin Hospital Ethics Committee, Shanghai Jiao-Tong University School of Medicine.

### Animals

*Men1*^*flox/flox*^ mice (129/SvJ)^13^ were bred with mice expressing Cre recombinase driven by the rat insulin promoter (*Rip-Cre*) to generate the βMen1-KO mice. *Ctnnb1*^*flox/flox*^ mice (C57BL/6J) were purchased from Jackson Laboratory. The βBcat-KO mice were generated by breeding *Ctnnb1*^*flox/flox*^ mice and *Rip-Cre* mice (mixed C57BL/6J:129/SvJ). Heterozygous (*Men1*^f/+^-*Cre*^+^, *Ctnnb1*^*f/*+^-*Cre*^+^) mice (mixed C57BL/6J:129/SvJ) were backcrossed with C57BL/6J mice five times and then crossed to generate homozygous mice. The βMen1/Bcat-KO mice (C57BL/6J) were generated by breeding βMen1-KO mice (C57BL/6J) and βBcat-KO mice (C57BL/6J). The C57BL/6J mice were purchased from the Shanghai Laboratory Animal Center, Chinese Academy of Sciences (SLAC, CAS). All of the mice were housed in pathogen-free facilities with a 12 h light/dark cycle and had free access to water and food. The βBcat-KO and control mice were fed a standard chow diet or a HFD (research diets) for 4 months. βMen1-KO mice, at the age of 14 months, were intraperitoneally injected with PKF115-584 (0.5 mg kg^−1^, Novartis Pharmaceuticals) or vehicle (0.2% dimethylsulphoxide in PBS) every 3 days for 8 weeks. All mice used for the experiments were males. All of the animal experiments were conducted in accordance with the Guide for the Care and Use of Laboratory Animals published by the National Institutes of Health.

### Isolation and culture of mouse pancreatic islets

Two millilitre of 1 mg ml^−1^ type XI collagenase (Sigma) in Hank’s buffered saline solution was injected into the pancreas of the mouse models through the bile duct. The pancreas was removed and incubated for 17 min at 37 °C and dissociated by mechanical pipetting. The islets were hand-picked under a dissecting microscope. The islets were cultured with RPMI 1640 (Invitrogen) culture medium supplemented with 10% (v/v) foetal bovine serum (Invitrogen), 100 U ml^−1^ penicillin and 100 μg ml^−1^ streptomycin (Invitrogen) for recovery in a sterile incubator at 37 °C with 5% CO_2_ infusion and humidified air. The islets were dissociated with a trypsin EDTA solution (Invitrogen) after overnight incubation and placed into poly-Lysine (Sigma) coated dishes.

### PNET cell preparation

PNET cells were isolated from βMen1-KO mice older than 8 months. Hank’s buffered saline solution (3 ml, Invitrogen) was injected into the pancreas of the mouse through the bile duct to facilitate distinguishing the tumours. The PNETs were detached under a dissecting microscope and minced on ice immediately. The minced tissues were dissociated with a trypsin EDTA solution for 30 s and passed through a Bellco Cellector (Bellco Biotechnology). The extruded cells were rinsed and cultured with RPMI 1640 culture medium supplemented with 10% (v/v) foetal bovine serum, 100 U ml^−1^ penicillin and 100 μg ml^−1^ streptomycin. The cells were treated with PKF115-584 (1 mM for 16 h) or vehicle. Cell proliferation assay was performed using EdU assay kit (Click-iT EdU Imaging Kit, Invitrogen). The dispersed cells were treated with EDU (10 μM) for 12 h before immunostaining. Positive cells were analyzed with an Olympus microscopy system.

### Cell culture and transfection

Human embryonic kidney 293T (HEK293T) cells were maintained in Dulbecco’s modified Eagle’s medium supplemented with 10% foetal calf serum at 37 °C in 5% CO_2_ infusion and humidified air. 293T cells were transfected with plasmids by Lipofectamine 2000 (Invitrogen). Human full-length *MEN1* complementary DNA was inserted in pCI-neo vector (Promega) to create the human *MEN1* expression construct. β-catenin plasmid with green fluorescent protein tag was obtained from Xiang Yu (Institute of Neuroscience, SIBS, CAS) with permission from James Nelson (Stanford University). MG132 (25 μM, Sigma) was used to treat transfected 293T cells for 2 h before harvest.

### Micro-PET/CT imaging and image processing

Micro-PET/CT was performed as described previously^44^. Briefly, the overnight fasted mice were injected with fluorine-18 fluorodeoxyglucose (7.4 MBq, 100 μl) via the tail vein. Micro-PET/CT (Inveon mPET/CT; Siemens Preclinical Solution, Knoxville, TN, USA) was performed 30 and 120 min after anaesthesia. Micro-PET imaging was reconstructed using the standard ordered-subset expectation maximization method. The standardized uptake values were obtained using the Invecon Acquisition Workplace (Siemens AG, Erlangen, Germany). The PET images were reconstructed using OSEM3D/MAP.

### Pancreatic histological and immunofluorescence analysis

The tissues were fixed in 4% paraformaldehyde for at least 24 h, dehydrated and paraffin embedded. Hematoxylin–eosin staining was performed on 4-μm sections. Immunofluorescence and immunohistochemical staining were performed according to the standard protocols. The following primary antibodies were used: anti-Ki67 antibody (1:400; Bethyl laboratories), anti-menin antibody (1:10,000; Bethyl laboratories), anti-β-catenin antibody (1:100; Cell Signalling Technology), anti-Mcm2 antibody (1:100; Cell Signalling Technology) and anti-insulin (1:3,000; Dako). The secondary antibodies for the immunofluorescence staining were purchased from Invitrogen, Dako and Jackson ImmunoResearch. The images were acquired using an Olympus microscopy system.

### β-cell mass

Whole pancreas from 4-month-old mice were weighed, fixed, embedded and sectioned throughout. A series of sections 250 μm apart (12–17 sections per sample) were immunostained with primary anti-insulin antibody (1:800; Cell Signalling Technology) followed by peroxidase-conjugated secondary antibody, visualized using a DAB Peroxidase Substrate Kit (Maixin) and counterstained with eosin. The whole area for all sections was imaged using Nikon Coolscan 9000 and NikonScan (version 4.0.2). The total pancreas area and insulin-positive area of each section were measured using MetaMorph version 6.1 (Molecular Devices). β-Cell mass (mg) was calculated by multiplying pancreata weight with the ratio of total insulin-positive area to total pancreas area in all sections.

### Glucose tolerance test and insulin tolerance test

The mice were fasted for 18 h before the glucose tolerance test, and they were fasted for 6 h before insulin tolerance test. The mice were injected intraperitoneally with either 2 g kg^−1^ glucose or 0.75 U kg^−1^ insulin (Regular Humulin, Eli Lilly and Company). The glucose measurements were taken up to 2 h post injection using One-Touch Ultra glucometers (LifeScan). The serum insulin levels were measured by a mouse insulin ELISA kit (Crystal Chem).

### Pancreatic perfusion

The pancreatic perfusion experiments were performed as previously described^45^. Briefly, the pancreata were isolated with segments of the duodenum and spleen. An arterial cannula was introduced into the coeliac artery, and a venous cannula was inserted into the portal vein. The perfusate consisted of Krebs-Ringer Bicarbonate (KRB) containing 2.8 or 16.7 mM glucose supplemented with 3% dextran (Sigma) and 1% BSA gassed with 95% O_2_/5% CO_2_. The perfusate was introduced into the coeliac artery at a rate of 1 ml min^−1^, and the effluent was collected at 1 min intervals from the portal vein after a single passage through the pancreas. The insulin levels in the perfusate were measured by ELISA.

### Islet transplantation

The STZ-induced diabetic mice were generated by single intraperitoneal injection (180 mg kg^−1^, Sigma) in 2-month-old C57BL/6 mice. Diabetic hyperglycaemia was defined as a nonfasting blood glucose concentration of >11.1 mmol l^−1^ on two or more consecutive days. The pancreatic islets of the βMen1-KO and βMen1/Bcat-KO mice were isolated for transplantation. Two hundred islet equivalents were transplanted beneath the kidney capsule of the diabetic recipients.

### Real-time reverse transcription–PCR

The total RNA was extracted using the RNeasy mini-kit (QIAGEN). For reverse transcription, 1 μg of the total RNA was converted into complementary DNA in a 20 μl reaction volume using a reverse transcription kit (Promega) following the manufacturer’s instruction. Quantitative RT–PCR was performed using a LightCycler 480 Real-Time PCR System (Roche Applied Science). The gene expression levels were normalized to the housekeeping gene *GAPDH* or 18S ribosomal RNA. Primer sequences can be found in the Supplementary Methods.

### Western blot

Protein preparation and Western blots were performed as described previously^15^. Nuclear and cytoplasmic lysates were prepared with the NE-PER kit (Pierce Biotechnology) according to the manufacturer’s instructions. The protein levels of active β-catenin in whole-cell lysates were quantified using the anti-Active β-catenin clone 8E7 antibody (Millipore). The antibody is specific for the active form of β-catenin, dephosphorylated on Ser37 or Thr41. The following primary antibodies were used: anti-menin (1:1,000; Cell Signalling Technology), anti-β-catenin (1:1,000; Cell Signalling Technology), anti-active β-catenin (1:1,000; Millipore), anti-Phospho-β-catenin (Ser33/37/Thr41; 1:1,000; Cell Signalling Technology), anti-Phospho-β-catenin (Ser45; 1:1,000; Cell Signalling Technology), anti-α-tubulin (1:1,000; Cell Signalling Technology), anti-α-tubulin (1:1,000; Cell Signalling Technology), anti-Cyclin D2 (1:1,000; Cell Signalling Technology), anti-Cyclin A (1:1,000; Abcam), anti-Cyclin B2 (1:1,000; Santa Cruz Biotechnology), anti-Mcm2 (1:1,000; Cell Signalling Technology), anti-Pbk (1:1,000; Cell Signalling Technology), anti-C-myc (1:1,000; Cell Signalling Technology), anti-GAPDH (1:10,000; KANGCHEN) and anti-lamin B (1:1,000; Santa Cruz Biotechnology). Images have been cropped for presentation. Full size images are presented in Supplementary Figs 9–11.

### ChIP assay

ChIP assays on PNETs and pancreatic islets were performed using an EZ ChIP kit (Upstate Biotechnology) according to the manufacturer’s protocol. Briefly, 1 × 10^6^ dispersed cells from the mouse models were cross-linked by 1% formaldehyde. For the PKF115-584 treatment, dispersed tumour cells were treated with PKF115-584 (1 mM for 16 h) or vehicle, followed by formaldehyde cross-linking. The cell lysates were sonicated to shear the chromatin into 200–1,000-bp fragments. Hundred micrograms of sheared chromatin and 4 μg of anti-β-catenin antibody (Cell Signalling Technology) or normal immunoglobulin-G (Cell Signalling Technology) were used for each immunoprecipitation. Quantitative RT–PCR was performed on the precipitated DNAs. Primer sequences can be found in the Supplementary Methods.

### Statistical analysis

Significant differences were analyzed using two-tail unpaired Student’s *t*-test or Fisher’s exact test. The error bars in the graphs represent s.d. Differences were considered significant if *P*<0.05.

## Author contributions

Y.Ca. and X.J. conceived and designed the experiments, contributed to research data and wrote the manuscript. F.L. contributed to research data; Y.S., Y.L., Y.P. and Y.Ch. contributed to mouse experiments; C.Z. contributed to Men1^*flox*/*flox*^ mouse models. W.W. contributed to discussion and reviewed the manuscript. G.N. designed the experiments, contributed to discussion, and reviewed and edited the manuscript.

## Additional information

**How to cite this article**: Jiang, X. *et al.* Targeting β-catenin signalling for therapeutic intervention in *MEN1*-deficient pancreatic neuroendocrine tumours. *Nat. Commun.* 5:5809 doi: 10.1038/ncomms6809 (2014).

## Supplementary Material

Supplementary Information Supplementary Figures 1-11 and Supplementary Methods.
