# Supplementary material for: Targeting β-catenin signaling for therapeutic intervention in MEN1-deficient pancreatic neuroendocrine tumours
Source: Nat Commun. 2014 Dec 17;5:5809. doi: 10.1038/ncomms6809 (PMC4284642; doi:10.1038/ncomms6809)
Supplement: Supplementary Information — Supplementary Figures 1-11 and Supplementary Methods. [file ncomms6809-s1.pdf]

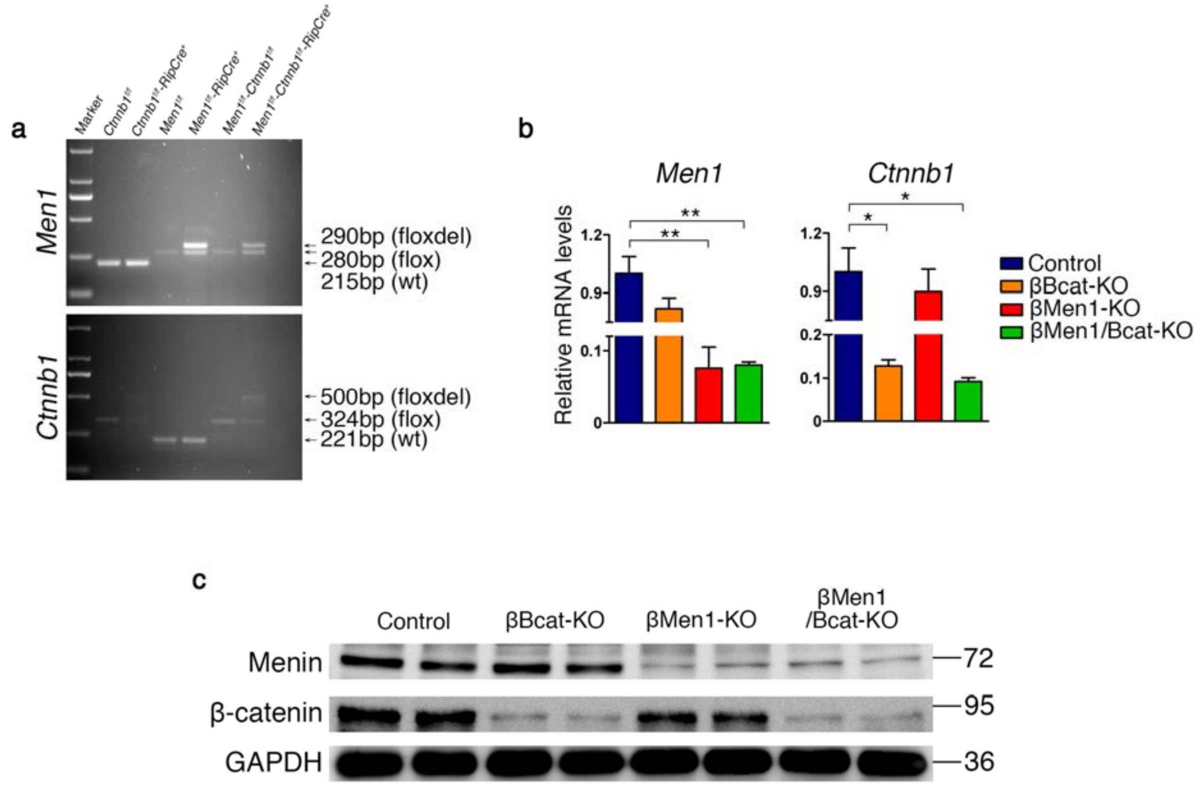

Supplementary Figure 1. Pancreatic β-cell specific knockout of *Men1* and *Ctnnb1*.

(a) Genotyping of the *Ctnnb1*$^{flox/flox}$, *Ctnnb1*$^{flox/flox}$-*RipCre*$^{+}$, *Men1*$^{flox/flox}$, *Men1*$^{flox/flox}$-*RipCre*$^{+}$, *Men1*$^{flox/flox}$-*Ctnnb1*$^{flox/flox}$ and *Men1*$^{flox/flox}$-*Ctnnb1*$^{flox/flox}$-*RipCre*$^{+}$ mice by PCR using genomic DNA from isolated islets. **(b)** qPCR analysis of *Men1* and *Ctnnb1* in isolated islets from 8-week-old mouse models (n = 3 for each group). The data represent the mean ± SD, *P < 0.05, **P < 0.01, Student's t-test. **(c)** Western blot analyses of menin and β-catenin in isolated islets from 8-week-old mouse models. The data shown represent three independent experiments.

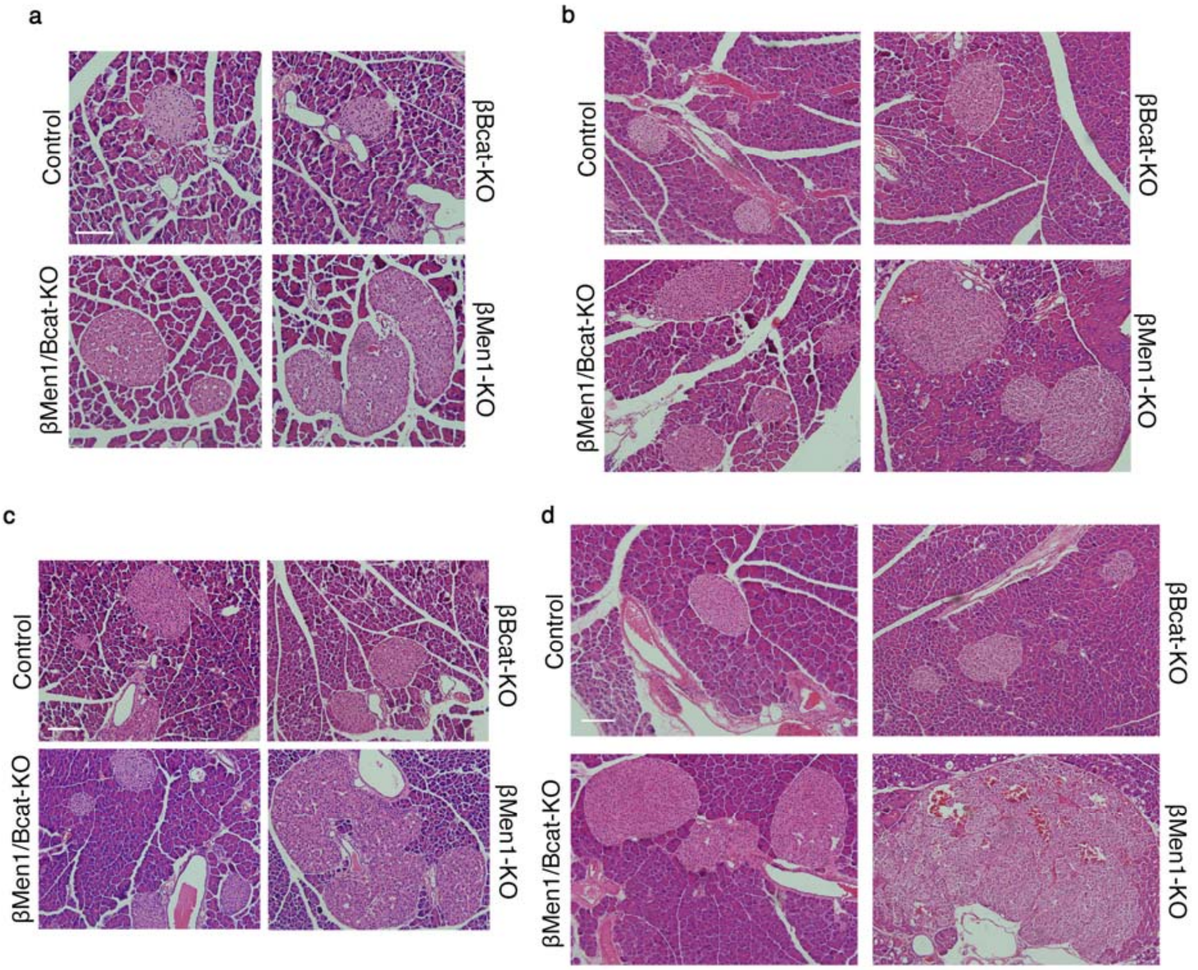

Supplementary Figure 2. H&E staining of pancreatic sections from mouse models at the age of 4 months (a), 6 months (b), 8 months (c) and 12 months (d). Scale bars, 100 μm.

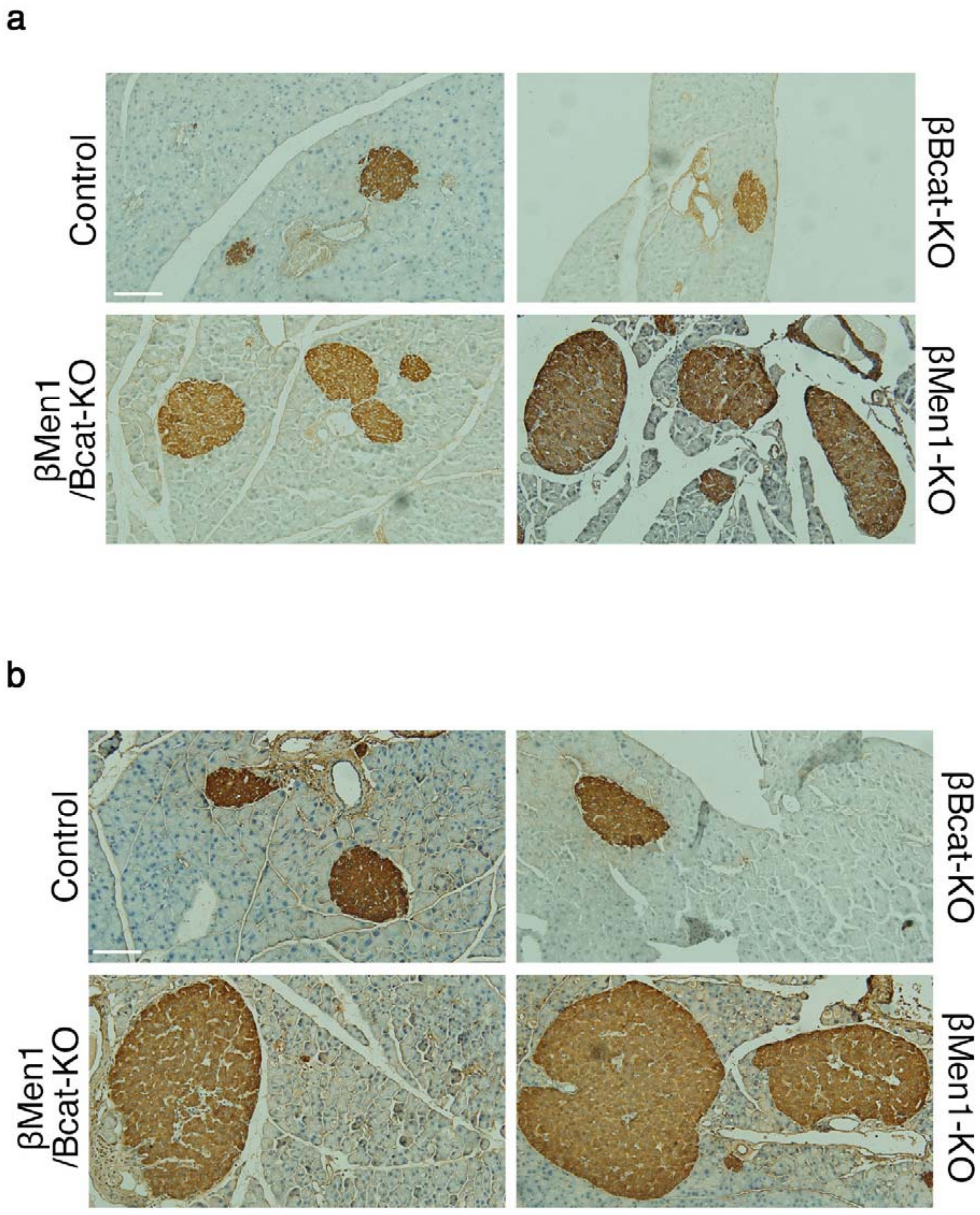

Supplementary Figure 3. Immunohistochemical staining of insulin on the pancreatic sections from 4-month-old (a) and 8-month-old (b) mouse models. Scale bars, 100 μm.

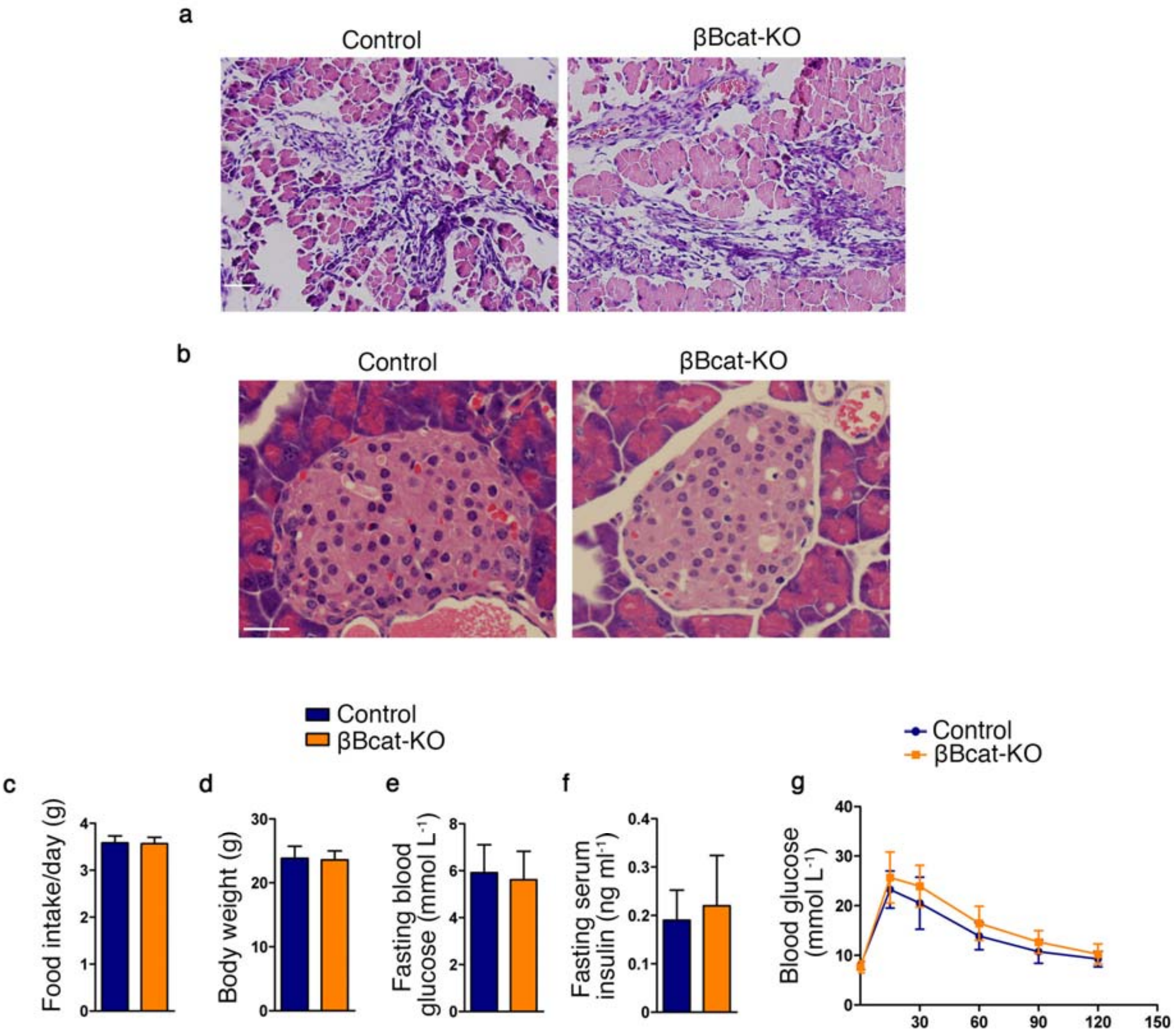

Supplementary Figure 4. The metabolic phenotypes of βBcat-KO mice on normal chow. **(a)** H&E staining of the pancreas from βBcat-KO and control mouse embryos at E18.5. **(b)** H&E staining of the pancreas from 8-week-old βBcat-KO mice. Scale bars, 25 µm. **(c)** Food intake of 12-week-old male βBcat-KO and control mice (*n* = 6). **(d-f)** Body weight **(d)**, fasting blood glucose levels **(e)** and fasting serum insulin levels **(f)** of 12-week-old βBcat-KO and control mice (*n* = 12). **(g)** Glucose tolerance test in 12 week-old βBcat-KO and control mice (*n* = 5-6). The data represent the mean ± s.d..

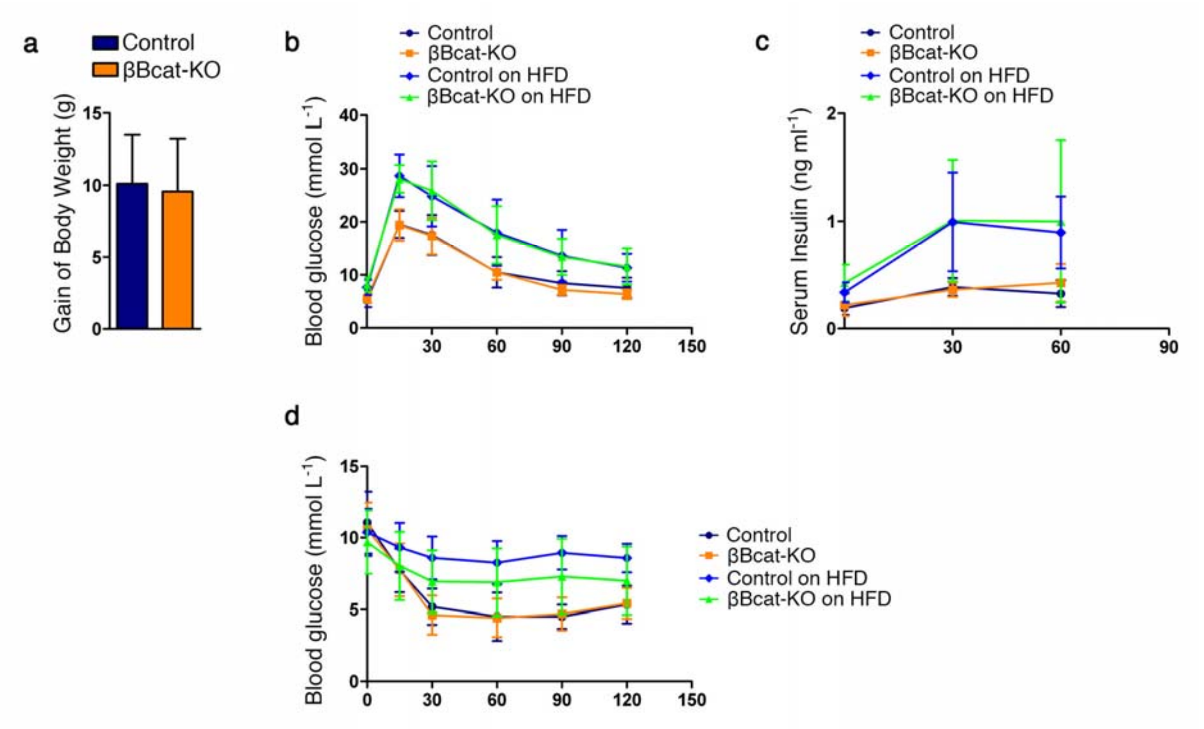

Supplementary Figure 5. Glucose metabolism of βBcat-KO mice on HFD.

(**a**) Body weight gain of βBcat-KO and control mice on HFD (n = 14). **(b)** Glucose tolerance test of 6-month-old βBcat-KO and control mice on HFD and normal chow (n = 11-15). **(c)** Serum insulin levels during the glucose tolerance test (n = 8-12). **(d)** Insulin tolerance test in βBcat-KO and control mice on HFD and normal chow (n = 10 for each group). The data represent the mean ± s.d..

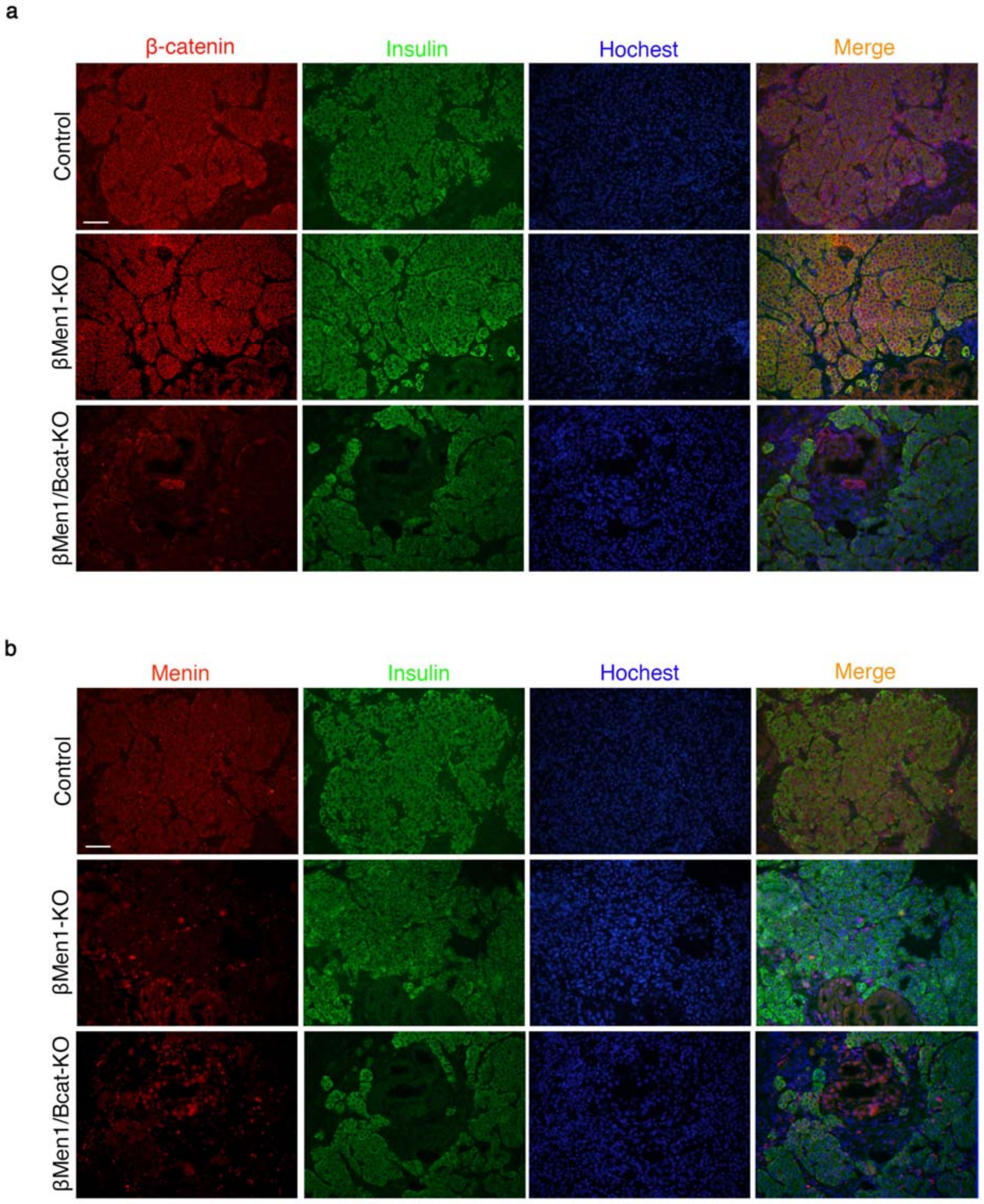

Supplementary Figure 6. Staining of menin and β-catenin in transplanted pancreatic islets. (a) Immunofluorescence staining of β-catenin and insulin on the sections of transplanted islets from control, βMen1-KO and βMen1/Bcat-KO mice. **(b)** Immunofluorescence staining of menin and insulin on the sections of transplanted islets from control, βMen1-KO and βMen1/Bcat-KO mice. Scale bars, 100 μm.

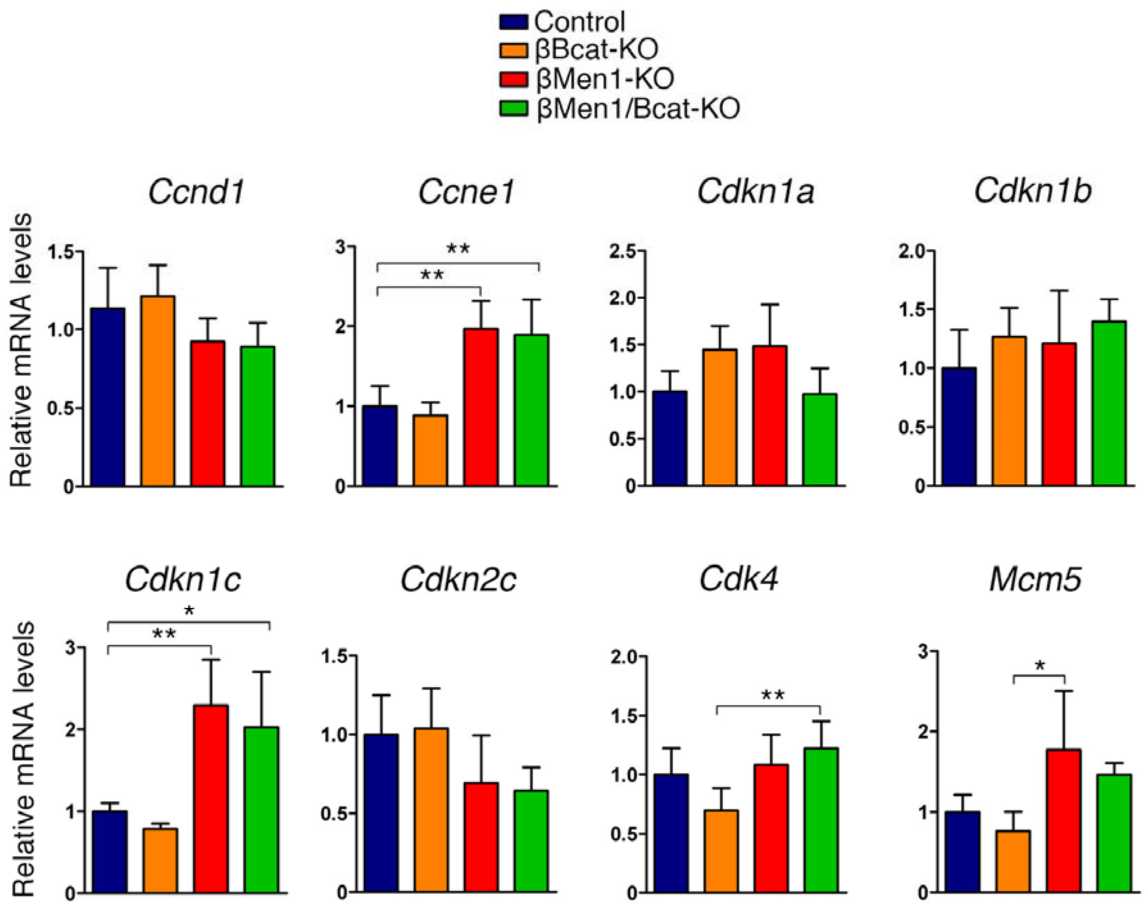

Supplementary Figure 7. qRT-PCR analysis. Expression levels of *Ccnd1*, *Ccne1*, *Cdkn1a*, *Cdkn1b*, *Cdkn1c*, *Cdkn2c*, *Cdk4* and *Mcm5* were analyzed (n = 4-6). The data represent the mean ± s.d., *P < 0.05, **P < 0.01, Student's t-test. The data shown represent three independent experiments.

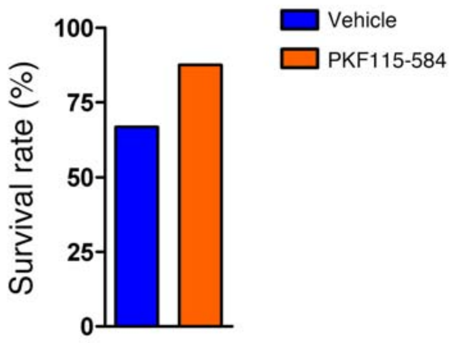

Supplementary Figure 8. Survival rate analysis. The survival rate of the βMen1-KO mice (*n* = 8-9) treated with PKF115-584 (0.5 mg kg^{-1}) or vehicle *in vivo* were analyzed.

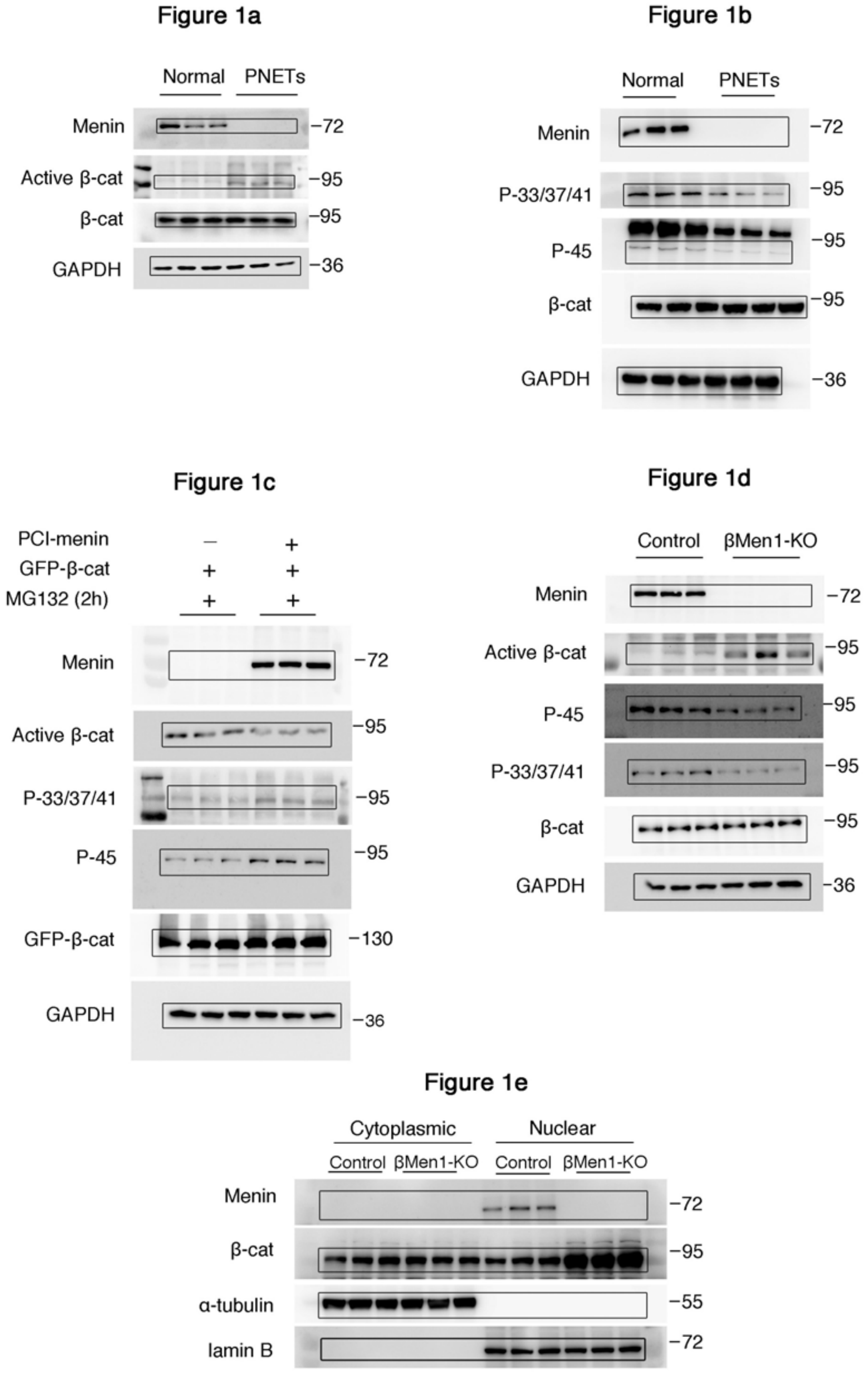

Supplementary Figure 9. Uncropped Western blots to the corresponding figures. Molecular weight makers are indicated in KDa.

Figure 6a

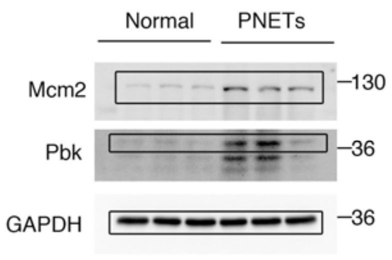

Figure 6c

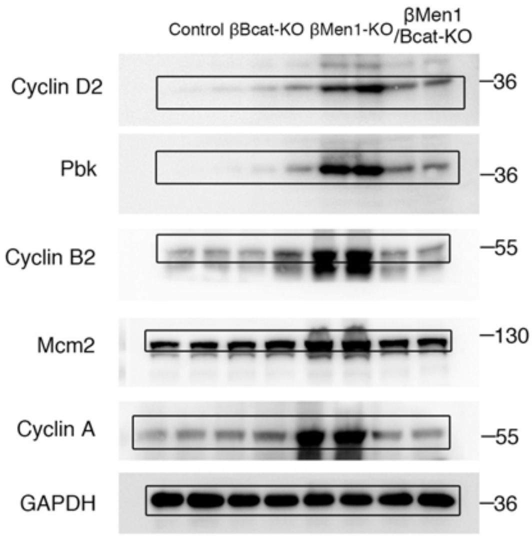

Figure 7f

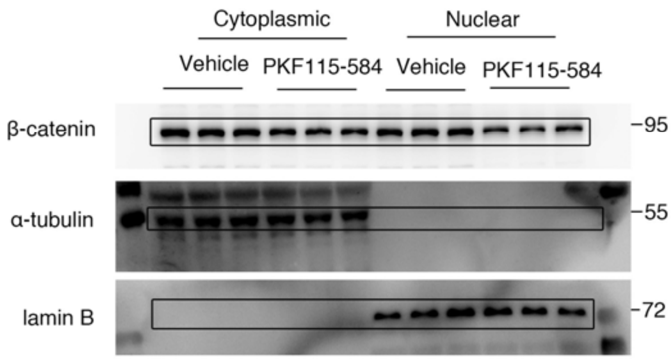

Figure 7i

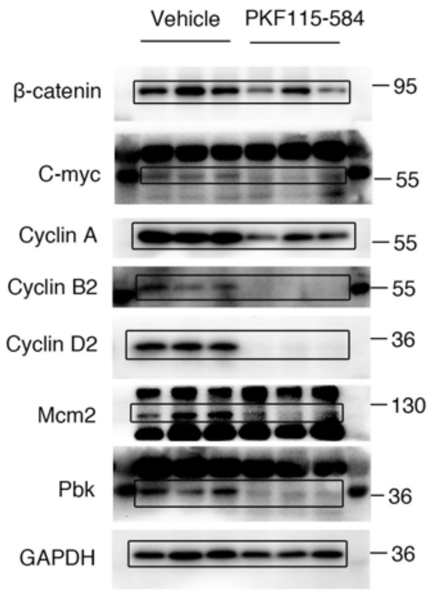

Supplementary Figure 10. Uncropped Western blots to the corresponding figures. Molecular weight makers are indicated in KDa.

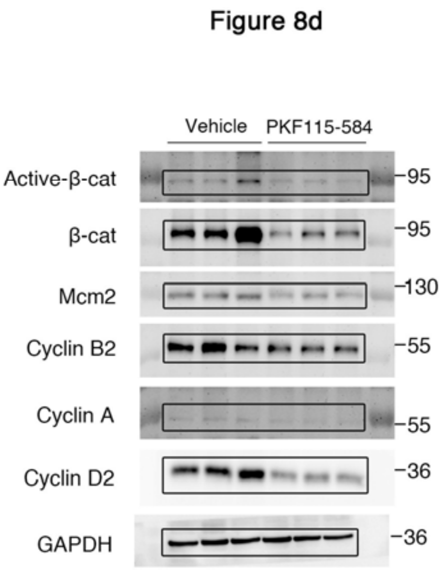

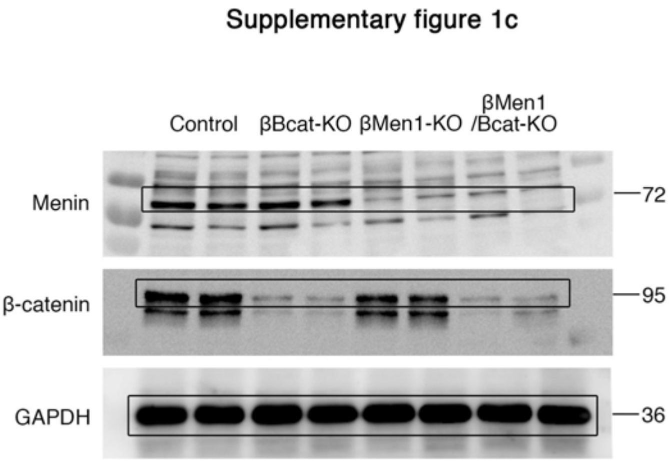

Supplementary Figure 11. Uncropped Western blots to the corresponding figures.

Molecular weight makers are indicated in KDa.

Supplementary Methods

Primers

The following primers for quantitative RT-PCR were used:

Gene	Species	Primer Sequence (5'-3')
Ccna2	Mouse	F: GCCTTCACCATTCATGTGGAT
		R: TTGCTCCGGGTAAAGAGACAG
Ccnb2	Mouse	F: AGCTCCCAAGGATCGTCCTC
		R: TGTCCTCGTTATCTATGTCCTCG
Ccnd1	Mouse	F: GCGTACCCTGACACCAATCTC
		R: ACTTGAAGTAAGATACGGAGGGC
Ccnd2	Mouse	F: GAGTGGGAACTGGTAGTGTTG
		R: CGCACAGAGCGATGAAGGT
Ccne1	Mouse	F: CTCCGACCTTTCAGTCCGC
		R: CACAGTCTTGTCAATCTTGGCA
Cdk4	Mouse	F: ATGGCTGCCACTCGATATGAA
		R: TGCTCCTCCATTAGGAACTCTC
Cdkn1a	Mouse	F: CCTGGTGATGTCCGACCTG
		R: CCATGAGCGCATCGCAATC
Cdkn1b	Mouse	F: TCAAACGTGAGAGTGTCTAACG
		R: CCGGGCCGAAGAGATTTCTG
Cdkn1c	Mouse	F: GCAGGACGAGAATCAAGAGCA
		R: GCTTGGCGAAGAAGTCGTT
Cdkn2c	Mouse	F: GGGGACCTAGAGCAACTTACT
		R: AAATTGGGATTAGCACCTCTGAG
Ctnnb1	Mouse	F: TCCCTGAGACGCTAGATGAGG
		R: CGTTTAGCAGTTTTGTCAGCTC
Mcm2	Mouse	F: AACTGTAGCAAGTGCAACTTTGT
		R: GCGGATACGTTGGTAGTTCTGAT
Mcm5	Mouse	F: TGAACTCAAGCGGCATTACAA
		R: GGCTGTTTATGCAAGTGGTCA
Mcm6	Mouse	F: CCTGAGAGAAACACGCTGGTT
		R: CGACACAGGTAAGGGTAGACTC
Men1	Mouse	F: TCATTGCTGCCCTCTATGCC
		R: TCCAGTTTGGTGCCTGTGATG
Mki67	Mouse	F: ATCATTGACCGCTCCTTTAGGT
		R: GCTCGCCTTGATGGTTCCT
Myc	Mouse	F: CCTAGTGCTGCATGAGGAGACA
		R: CCTCATCTTCTTGCTCTTCTTCAGA
Pbk	Mouse	F: ACTGGGGTCAGCGTTTACCTA
		R: GCACAGACTACCATCACTGGC
Rn18s	Mouse	F: ACCGCAGCTAGGAATAATGGA
		R: GCCTCAGTTCCGAAAACCA
CCNA2	Human	F: CGCTGGCGGTACTGAAGTC
		R: GAGGAACGGTGACATGCTCAT
CCNB2	Human	F: TGCTCTGCAAAATCGAGGACA

		R: GCCAATCCACTAGGATGGCA
CCND2	Human	F: TTTGCCATGTACCCACCGTC
		R: AGGGCATCACAAGTGAGCG
GAPDH	Human	F: ACAACTTTGGTATCGTGGAAGG
		R: GCCATCACGCCACAGTTTC
MCM2	Human	F: ATGATCGAGAGCATCGAGAACC
		R: GCCAAGTCCTCATAGTTCACCA
MKI67	Human	F: GCCTGCTCGACCCTACAGA
		R: GCTTGTCAACTGCGGTTGC
PBK	Human	F: CCAAACATTGTTGGTTATCGTGC
		R: GGCTGGCTTTATATCGTTCTTCT
PCNA	Human	F: CCTGCTGGGATATTAGCTCCA
		R: CAGCGGTAGGTGTCGAAGC

The following primers for ChIP assays were used:

**Primer Name**	**Amplicon site**	**Primer Sequence (5'-3')**
Ccnd1-p1	-149 − 63	F: CCCAGTTTGGAGAGAAGCAG
		R: ACTCCCCTGTAGTCCGTGTG
Ccnd1-p2	-660 − -400	F: CCAGCGAGGAGGAATAGATG
		R: AGCGTCCCTGTCTTCTTTCA
Ccnd1-p3	-1044 − -791	F: TCACCTTATCGGCTCACAAGT
		R: AGACACGATAGGCTCCTTCC
Ccnd1-p4	-2750 − -2552	F: TGAAATCCGCTCAGGGTAAC
		R: GGACTTGGCTGTTTCTGCTC
Myc-p1	-288 − -35	F: ATACGCAGGGCAAGAACACAG
		R: TTTTTTCCTCCTCTCGCTTCC
Myc-p2	-437 − -329	F: GTTCGTCCTTCCCCCTTTCTAA
		R: GCTCCACACAATACGCCATGTA
Myc-p3	-1030 − -885	F: TCATGGCATATTCTCGCGTCTA
		R: CCCCATACACCTCCACACAGTT
Myc-p4	-1492 − -1320	F: TGTAGGATAAGCAAATCCCGAGG
		R: TCCTGAATACTACGCTGTGCATTC
Mcm2-p1	-221 − -101	F: GGCCATACCGACACTTAGATACCT
		R: GCTTAACCCTTCAGCGTTTACCA
Mcm2-p2	-485 − -308	F: GTGCTTGAAGGATTGAACCTCAGT
		R: GCTGTCCTGGAACTCACTCTGT
Mcm2-p3	-712 − -585	F: AGCTCCAACTCCTAGACAGACTG
		R: CGTGATGCAGTACAGCGAAGG
Mcm2-p4	-1232 − -1012	F: GGCTTATGGTGGACACAGTCAG
		R: AGGGTATCTTAGGGAGATGGGCTA
Mcm2-p5	-1428 − -1326	F: AGGCTACTTGGACTCTTCACAGAA
		R: CTCTCATTACTGGTCTGCCATTCC
Mcm2-p6	-1579 − -1335	F: ATGTCAATCTCACTCGCTGTTCTG
		R: CTGGTCTGCCATTCCATGCTTAC
